# Characterization of *Enterobacter cloacae complex* clinical isolates: comparative genomics and the role of the efflux pump AcrAB-TolC over-expression and NDM-1 production

**DOI:** 10.3389/fcimb.2025.1705370

**Published:** 2025-11-07

**Authors:** Nan Jiang, Faxiao Pu, Wenji Wang, Dongguo Wang, Jiayu He

**Affiliations:** 1 Department of Integrated Chinese and Western Medicine, Taizhou Municipal Hospital Affiliated to Taizhou University, Taizhou, Zhejiang, China; 2 Department of Clinical Laboratory Medicine, Zhangye People’s Hospital Affiliated to Hexi University, Zhangye, Gansu, China; 3 School of Life Sciences, Taizhou University, Taizhou, Zhejiang, China; 4 Department of Central Laboratory, Taizhou Municipal Hospital Affiliated to Taizhou University, Taizhou, Zhejiang, China; 5 Department of Clinical Laboratory Medicine, Jiaojiang Maternal and Child Health Hospital, Taizhou, Zhejiang, China

**Keywords:** carbapenem-resistant Enterobacter cloacae complex (ECC), AcrAB-TolC efflux pump, blaNDM-1 carbapenemase, acrAB-tolC over-expression, the blaNDM-1-bleMBL module, Tn125/Tn3000 transposons

## Abstract

**Background:**

Carbapenem-resistant Enterobacter cloacae complex (ECC) has emerged as the third most prevalent nosocomial Enterobacterales pathogen, propelled by the synergy between intrinsic defenses, e.g. AcrAB-TolC efflux pump overexpression and horizontally acquired determinants such as the plasmid-borne *bla*_NDM-1_.

**Methods:**

Our study employed a comprehensive, multi-faceted strategy to characterize three carbapenem-resistant, NDM-1-positive ECC isolates, named x9, F12, and x230151. The integrated methodology merged phenotypic antimicrobial susceptibility testing with functional conjugation and electroporation assays. High-resolution hybrid Nanopore-Illumina genome assemblies enabled detailed genomic annotation and extensive resistance profiling. We further quantified *acrAB-tolC* efflux pump expression via quantitative RT-PCR and reconstructed the evolutionary history of the *bla*_NDM-1_-*ble*_MBL_ module using comparative plasmid genomics.

**Results:**

All three ECC isolates were extensively Drug-Resistant (XDR) with carbapenem MICs ≥ 8 mg/L. Genomes carried 33–35 resistance loci, including > 5-fold up-regulated *acrAB-tolC* on chromosome, and constitutive chromosomal expression of *bla*_ACT_-type AmpC further blunted the activity of cephalosporins and carbapenems. The *bla*_NDM-1_-*ble*_MBL_ resided on ~110–240 kb IncF/IncN/IncX3 plasmids that transferred at 3-8×10–^3^ per donor and were embedded in Tn*125*/Tn*3000* transposons flanked by IS*Aba125*/IS*26*. These replicons also carried ESBLs (*bla*_CTX-M-15_), *bla*_TEM-1_, aminoglycoside- modifying enzymes (*armA*, *aadA1*) and intact class 1 integrons (In2, In799 and In1465). Despite>90% backbone identity of the *bla*_NDM-1_-*ble*_MBL_, the module could be reversibly excised, inverted or truncated, allowing rapid “gene-offload” and re-acquisition under shifting antibiotic pressure.

**Conclusion:**

Once the carbapenem MIC surpassed 8 mg/L, the tandem action of the chromosomally over-expressed AcrAB-TolC efflux pump and plasmid-borne *bla*_NDM-1_ carbapenemase rendered ECC virtually untreatable. Mutations in *ramR* and *soxR* regulators, as well as promoter insertions or deletions that amplify either system should also be tracked in real-time via sustained surveillance.Effective containment would require combination strategies that simultaneously inhibit efflux, neutralize β-lactamase activity, and destabilized or eliminated the IncF/IncN/IncX3 plasmids carrying *bla*_NDM-1_.

## Introduction

The *Enterobacter cloacae complex* (ECC) ranks among the foremost nosocomial pathogens, repeatedly seeding bloodstream infections, ventilator-associated pneumonia, catheter-related urinary- tract infections, and surgical-site infections - particularly in the immunocompromised. Over the past decade, the global dissemination of extended-spectrum β-lactamases (ESBLs) and carbapenemases (NDM, KPC) had catapulted ECC to the position of the third most prevalent Enterobacteriaceae driving hospital-acquired infections, trailing only *Escherichia coli* and *Klebsiella pneumoniae* (Davin-Regli and Pagès, 2025).

As shown previously, the AcrAB-TolC was the dominant RND-type efflux apparatus in *E. cloacae* (Davin-Regli and Pagès, 2025). By forming a tripartite conduit spanning the inner membrane (AcrB), periplasm (AcrA), and outer membrane (TolC) and harnessing the proton-motive force, this pump extruded a chemically diverse arsenal of antibiotics, fluoroquinolones, tetracyclines, chloramphenicol, and tigecycline, thereby attenuating intracellular drug levels and driving phenotypic resistance (Davin-Regli and Pagès, 2025). AcrAB-TolC over-expression was documented in approximately 40% of multidrug resistance clinical isolates, underscoring its pivotal role in phenotypic resistance, and transcription was amplified by the global activators RamA, MarA, and SoxS, while the local repressor AcrR exerts a braking effect, collectively fine-tuning efflux capacity in response to environmental cues (Chirabhundhu et al., 2024).

The *bla*_NDM-1_ gene encoded a Zn^2+^- dependent carbapenemase that hydrolysed virtually all β- lactam scaffolds, including the “last-line” carbapenems meropenem, imipenem and ertapenem (Zhao et al., 2023). Isolates of the ECC that acquired *bla*_NDM-1_ routinely display meropenem and imipenem MICs >32 mg/L, relegating them to the extensively drug-resistant (XDR) category. Salvage therapy was then restricted to ceftazidime-avibactam or cefiderocol, yet even these last-resort agents lose reliability when the *bla*_NDM-1_ was partnered with the OXA-48-like carbapenemases or efflux-driven resistance (Zhao et al., 2023). Consequently, bloodstream infections caused by NDM-1-positive ECC carried 14-day mortality rates of 31-48%, roughly twice that observed with carbapenem-susceptible counterparts (Zhao et al., 2023). The *bla*_NDM-1_ was nested within the composite transposon Tn*125* and was routinely mobilized by broad-host-range plasmids of the IncF, IncA/C and IncX3 families (Zhao et al., 2023). These megaplasmids, often >200 kb, assembled a formidable resistance arsenal, encoding 16 S rRNA methylases (*armA*, *rmtB*) and ESBLs (*bla*_CTX-M-15_) that collectively forged multidrug-resistant core backbones. Conjugation efficiencies of ~10–^3^ transconjugants per donor cell, reinforced by the clonal dissemination of high-risk sequence types (ST78, ST114, ST171), exemplified the potent synergy of plasmid-driven horizontal transfer and epidemic clonal expansion on a global scale (Zhao et al., 2023). During the past decade, carbapenem-resistant ECC had emerged as a critical nosocomial pathogen. Population-based surveillance in China showed an upward trend in carbapenem non-susceptibility, with rates increasing from 5.5% in 2011 to 18.3% in 2019 (Wang et al., 2018).

In summary, the synergy between intrinsic defenses, exemplified by the potent AcrAB-TolC efflux pump, and horizontally acquired determinants such as the *bla*_NDM-1_ carbapenemase, had transformed ECC into a tenacious clinical adversary that continually outmaneuvered our antibiotic armamentarium. Despite considerable advances in ECC research, key gaps remain: no study has simultaneously dissected the relative contributions of the chromosomal *acrAB-tolC* efflux cluster and plasmid-borne *bla*_NDM-1_ within the same ECC isolates; current datasets are dominated by outbreak strains, under-representing sporadic and community isolates; and functional links between quantitative efflux expression, carbapenemase activity and clinical outcome are lacking. By unravelling the interplay between intrinsic efflux and acquired carbapenemase, this study will deliver actionable insights for surveillance, diagnostics and stewardship against this recalcitrant pathogen.

## Materials and methods

### Bacterial strains, comprehensive detection of β-lactamases and antimicrobial susceptibility testing

#### Bacterial strains

Six ECC isolates, F12, F43, X9, X220589, X220702, SC67, and X230151, collected between 2022 and 2023 were randomly retrieved from our hospital’s frozen archive for a preliminary carbapenemase screen. Isolates had been cryopreserved at -80°C in brain-heart-infusion broth supplemented with 15% (v/v) glycerol. After overnight revival on 5% sheep-blood agar at 35°C in ambient air, 2–3 well- isolated colonies from each isolate were suspended in 300 µL of 0.9% saline and tested by colloidal- gold immunochromatography (Fosun Diagnostics, Shanghai, China). The five-channel lateral-flow strips simultaneously detect KPC, NDM, IMP, VIM and OXA (**Table 1**). NDM-positive isolates, F12, x9, and x230151, will be investigated further in the study.

**Table 1:** Molecular characteristics of the six *E. cloacae* complex isolates.

Strain	Year of isolation	Ward	Specimen	MLST	Carpapenemase type	Beta- lactamase
F12	2020-01 (Taizhou, China)	Urinary surgery	*Sputum*	ST93	B (NDM)	A+B+C+D
F43	2020-06 (Taizhou, China)	Urinary surgery	Sputum	ST93	B (IMP)	A+B+C
x9	2024-08 (Taizhou, China)	Respiratory & Critical Care Medicine	Blood	ST116	B (NDM)	A+B+C
x220589	2023-10 (Taizhou, China)	ICU	Sputum	ST252	A (KPC)	A+C
x220702	2023-10 (Taizhou, China)	ICU	Sputum	ST171	None	C
x230151	2024-09 (Taizhou, China)	Respiratory & Critical Care Medicine	Urine	ST78	B (NDM)	A+B+C

#### Comprehensive carbapenemase detection: ambler classes A, B, and D

Detection of Ambler class A, B and D carbapenemases followed CLSI M100-Ed32 (Clinical and Laboratory Standards Institute (CLSI), 2022). A tiered algorithm combined inhibitor disks with a rapid biochemical screen. (1) Modified carbapenem- inactivation method (mCIM): A 10-µg meropenem disk was placed on Mueller-Hinton agar seeded with the test isolate; flanking disks contained 3 mg phenylboronic acid (PBA, class A inhibitor) or 292 µg EDTA (class B inhibitor). A ≥5-mm enlargement of the inhibition zone around the inhibitor disk versus the drug-alone disk defined PBA-sensitive (class A) or EDTA-sensitive (class B) carbapenemase activity (Wang et al., 2024). (2) EDTA-carbapenem inactivation method (eCIM): Performed as mCIM but with EDTA (292 µg) only; a zone ≥5 mm larger than the corresponding mCIM zone confirmed a metallo-β-lactamase (class B) (Workneh et al., 2019). (3) Modified Carba NP test (Dortet et al., 2012) with minor adjustments (Kumar et al., 2018): Four to five 10-µL loops of fresh colonies from blood agar were suspended in 400 µL Tris-HCl lysis buffer and vortexed (1 min). 100 µL of lysate was added to four 0.5-mL tubes: Tube 1 (negative control), 100 µL phenol red + 0.1 mM ZnSO_4_; Tube 2, Tube 1 + 6 mg/mL imipenem-cilastatin (≈3 mg/mL imipenem); Tube 3, Tube 2 + 4 mg/mL tazobactam; Tube 4, Tube 2 + 3 mM EDTA. Post-incubation, red→yellow shift in tubes 2 + 4 alone indicated class A; shift in tubes 2 + 3 indicated class B (MBL). No standardized phenotypic test exists for class D carbapenemases; their presence is inferred by exclusion when carbapenem resistance persists after inhibiting class A and class B enzymes (Wise et al., 2024).

#### Comprehensive β-lactamase detection: ESBL and ambler class C

ESBL confirmation (Clinical and Laboratory Standards Institute (CLSI), 2022): ≥5-mm increased in zone diameter for ceftazidime (CAZ, 30 µg) versus (vs) ceftazidime/clavulanic acid (CAZ/CLA, 30/10 µg) or cefotaxime (CTX, 30 µg) vs cefotaxime/clavulanic acid (CTX/CLA, 30/10 µg). Screening plate: 20/10 µg amoxicillin/clavulanic acid (AMC) disk centered; CTX and cefotaxime (CTX) (30 µg each) placed 25 mm away, cefepime (FEP, 30 µg) and cloxacillin (CXC, 200 µg) 20 mm away. Interpretation: AMC-CTX/CRO synergy = ESBL positive; no AMC-CTX/CRO synergy but AMC-FEP synergy = ESBL positive + AmpC; CXC- CTX/FEP synergy = AmpC.

Crude bete-lactamase extract preparation (Coudron et al., 2000): Overnight blood-agar growth was suspended to 0.5 McFarland; 25 µL was added to 6 mL Tryptic Soy Broth (TSB) and incubated (35°C, 200 rpm, 18 h). The culture was pelleted (4–000 rpm, 4°C, 20 min), subjected to five freeze-thaw cycles (-80°C), resuspended in 1.5 mL 0.01 M phosphate buffer (pH 7.0), and clarified (10, 000 rpm, 4°C, 15–30 min). The resulting enzyme extract was stored at -20°C until use. 3-D AmpC assay: 0.5 mL of 0.5 McFarland suspension was evenly spread on MH agar. A 30 µg cefoxitin disk was centered, and a 5 mm radial slit cut 5 mm from its edge. The slit was filled with 40 µL enzyme extract; after overnight incubation at 35°C, any indentation of the inhibition zone indicated AmpC production.

#### Antimicrobial susceptibility testing

Antimicrobial susceptibility testing was performed on the VITEK 2 system (bioMérieux) and verified by broth microdilution; minimum inhibitory concentrations (MICs) are summarized in **Table 2**. All tests were interpreted and validated according to CLSI M100-Ed32 [Clinical and Laboratory Standards Institute (CLSI), 2022)], with *E. coli* ATCC 25922 serving as the quality-control strain. MIC values were analyzed against CLSI breakpoints to determine resistance profiles [Clinical and Laboratory Standards Institute (CLSI), 2022)].

**Table 2:** Analysis of the genetic characteristics of *E. cloacae* strains x9, F12, and x230151, and comparison of their MICs with those of strain EC600 and its transconjugants.

Antimicrobial agents	MIC (mg/L)							Localization of the antibiotic-resistance gene in strains x9, F12, and x230151		
	EC600	x9	x9- EC600	F12	F12- EC600	x230151	x230151- EC600	x9	F12	x230151
Aminoglycoside										
Amikacin	≤2.0/S	≥64.0/R	≥8.0/R	≥64.0/R	≥16.0/R	≤2.0/S	≤2.0/S	*aadA2* (p1)	*armA* (p2), *armA*/*aadA1*/ *aac(3)-IId*/*aac(6')-Ib10*/ *aph(3')-Ia* (p3), *aph(3'')-Ib*/*aph(6)-Id* (p5)	None
Tobramycin	≤1.0/S	≥32.0/R	≥4.0/R	≥32.0/R	≥8.0/R	≤1.0/S	≤1.0/S			
β-lactams										
Ertapenem	≤0.064/S	≥8.0/R	≥2.0/R	≥8.0/R	≥2.0/R	≥8.0/R	≥2.0/R	*bla*_SHV-12_/*bla*_NDM-1_ (p1) , *bla*_ACT-17_/*PBP3* (Chr)	*bla*_LAP-2_ (p1), *bla*_TEM-1_/*bla*_NDM-1_ (p2), *bla*_TEM-1_/*bla*_SFO-1_ (p3), *bla*_ACT-17_/*PBP3* (Chr)	2*bla*_TEM-1_/*bla*_CTX-M-3_ (p1), *bla*_NDM-1_ (p2), *bla*_ACT-24_/*PBP3* (Chr)
Imipenem	≤0.125/S	≥16.0/R	≥4.0/R	≥16.0/R	≥4.0/R	≥16.0/R	≥4.0/R			
Meropenem	≤0.125/S	≥16.0/R	≥4.0/R	≥16.0/R	≥4.0/R	≥16.0/R	≥4.0/R			
Doripenem	≤0.125/S	≥8.0/R	≥2.0/R	≥8.0/R	≥2.0/R	≥8.0/R	≥2.0/R			
Aztreonam	≤0.25/S	≥64.0/R	≥16.0/R	≥64.0/R	≥16.0/R	≥64.0/R	≥16.0/R	*bla*_SHV-12_/*bla*_NDM-1_ (p1), *bla*_ACT-17_/*PBP3*/*acrAB-tolC* (Chr)	*bla*_LAP-2_ (p1), *bla*_TEM-1_/*bla*_NDM-1_ (p2), *bla*_TEM-1_/*bla*_SFO-1_ (p3), *bla*_ACT-17_/*PBP3*/*acrAB-tolC* (Chr)	2*bla*_TEM-1_ /*bla*_CTX-M-3_ (p1), *bla*_NDM-1_ (p2) , *bla*_ACT-24_/*PBP3*/*acrAB-tolC* (Chr)
Piperacillin	≤4.0/S	≥128.0/R	≥32.0/R	≥128.0/R	≥32.0/R	≥128.0/R	≥32.0/R			
Cefuroxime	≤8.0/S	≥64.0/R	≥16.0/R	≥64.0/R	≥16.0/R	≥64.0/R	≥16.0/R			
Ceftizoxime	≤4.0/S	≥64.0/R	≥16.0/R	≥64.0/R	≥16.0/R	≥64.0/R	≥16.0/R			
Ceftazidime	≤2.0/S	≥64.0/R	≥16.0/R	≥64.0/R	≥16.0/R	≥64.0/R	≥16.0/R			
Ceftriaxone	≤1.0/S	≥64.0/R	≥16.0/R	≥64.0/R	≥16.0/R	≥64.0/R	≥16.0/R			
Cefotaxime	≤1.0/S	≥64.0/R	≥16.0/R	≥64.0/R	≥16.0/R	≥64.0/R	≥16.0/R			
Cefepime	≤1.0/S	≥64.0/R	≥16.0/R	≥64.0/R	≥16.0/R	≥64.0/R	≥16.0/R			
Cefpodoxime	≤1.0/S	≥8.0/R	≥4.0/R	≥8.0/R	≥4.0/R	≥8.0/R	≥4.0/R			
Amoxicillin/ clavulanic acid	≤8.0/S	≥32.0/R	≥16.0/R	≥32.0/R	≥16.0/R	≥32.0/R	≥16.0/R			
Piperacillin/ Tazobactam	≤4.0/S	≥128.0/R	≥32.0/R	≥128.0/R	≥32.0/R	≥128.0/R	≥32.0/R			
Ceftazidime/ Avibactam	≤2.0/S	≥16.0/R	≥4.0/R	≥16.0/R	≥8.0/R	≥16.0/R	≥8.0/R			
Cefoperazone/ Sulbactam	≤2.0/S	≥64.0/R	≥16.0/R	≥64.0/R	≥16.0/R	≥64.0/R	≥16.0/R			
Cephamycin										
Cefoxitin	≤4.0/S	≥64.0/R	≥16.0/R	≥64.0	≥16.0/R	≥64.0	≥16.0/R	*bla*_SHV-12_/*bla*_NDM-1_ (p1), *bla*_ACT-17_/*PBP3* (Chr)	*bla*_LAP-2_ (p1), *bla*_TEM-1_*/bla*_NDM-1_ (p2), *bla*_TEM-1_/*bla*_SFO-1_ (p3), *bla*_ACT-17_/*PBP3* (Chr)	2*bla*_TEM-1_ /*bla*_CTX-M-3_ (p1), *bla*_NDM-1_ (p2), *bla*_ACT-24_/*PBP3* (Chr)
Fluoroqinolone										
Levofloxacin	≤0.5/S	≥2.0/R	≥1.0/R	≥8.0/R	≥2.0/R	≥8.0/R	≥2.0/R	*qnrB19* (p3), *acrAB-tolC* (Chr)	*qnrS1* (p1), *acrAB-tolC*/*gyrA*/*parC* (Chr)	*qnrA1* (p2), *acrAB-tolC*/*gyrA*/*parC* (Chr)
Ciprofloxacin	≤0.25/S	≥1.0/R	≥0.5/R	≥4.0/R	≥1.0/R	≥4.0/R	≥1.0/R			
Moxifloxacin	≤0.5/S	≥2.0/R	≥2.0/R	≥8.0/R	≥2.0/R	≥8.0/R	≥2.0/R			
Tetracycline										
Tetracycline	≤2.0/S	≥8.0/R	≥4.0/R	≥16.0/R	≥4.0/R	≥16.0/R	≥4.0/R	*acrAB-tolC* (Chr)	*acrAB-tolC* (Chr)	*acrAB-tolC* (Chr)
Glycylcycline										
Tigecycline	≤0.125/S	≥8.0/R	≥4.0/R	≥16.0/R	≥4.0/R	≥8.0/R	≥4.0/R	*acrAB-tolC* (Chr)	*acrAB-tolC* (Chr)	*acrAB-tolC* (Chr)
Sulfonamide										
Trimethoprim- sulfamethoxazole	≤20.0/S	≥160.0/R	≥80/R	320.0/R	80/R	≥320.0/R	≥80/R	*sul1* (p1)	*sul1* (p2), *sul1* (p3), *sul2* (p5)	*sul1*/*dfrA12* (p2)

Chr, chromosome; p, plasmid.

### Conjugate transfer and plasmid transfer experiments

#### Conjugation experiments

For conjugation assays, recipient *E. coli* EC600 and donor isolates, F12, x9, and x230151, were each grown to mid-logarithmic phase (OD600 0.5-0.7) in Luria-Bertani (LB) broth at 37°C, 200 rpm, the growth stage previously demonstrated to maximize plasmid transfer (Headd and Bradford, 2020). Equal volumes (0.5 mL each) of donor and recipient cultures were combined in 4 mL fresh LB broth to yield a 1:1 donor-to-recipient ratio. The mixtures were incubated statically at 35°C for 18–24 h to permit mating. After incubation, cells were plated on tryptic soy agar (TSA) supplemented with 10 µg/mL rifampicin and 0.02 µg/mL imipenem to select for transconjugants. Plates were incubated at 37°C for 18–24 h; colonies that arose were enumerated and presumed to be transconjugants. Successful plasmid transfer was subsequently verified by PCR and antibiotic- susceptibility profiling.

A negative-control mating was performed with *E. cloacae* ATCC 13047 as the donor and *E. coli* EC600 as the recipient under identical conditions. No colonies grew on selective plates, confirming that the antibiotic concentrations effectively suppressed background growth and that colonies observed in experimental matings represented genuine transconjugants.

#### Plasmid DNA extraction

Plasmid DNA was extracted from isolates, F12, x9, and x230151, with the Qiagen DNeasy Blood & Tissue Kit according to the manufacturer’s protocol. Overnight cultures (12–16 h) grown to log phase were pelleted at 4, 000 × g, 4°C, for 5 min. Pellets were washed once with ice-cold LB to remove residual antibiotics (Erdogan et al., 2017), followed by two washes with an equal volume of ice-cold Milli-Q water and centrifugation at 4°C. Washed cells were resuspended in 1/50 volume of 10% (v/v) glycerol, aliquoted, and stored at -70°C for downstream electroporation.

DNA concentration and purity were determined with a NanoDrop ND-1000 spectrophotometer, and quantity was confirmed using the Qubit dsDNA HS Assay Kit. Quality was assessed by electrophoresis: 1 µL of each sample was run on a 1% (w/v) agarose gel in TAE buffer, stained with ethidium bromide, and visualized under UV light to verify high-molecular-weight, undegraded DNA.

#### Plasmid electroporation

Electro-competent *E. coli* aliquots were mixed with ≤ 10 ng plasmid DNA in a chilled 0.2 cm cuvette and pulsed at 2.5 kV, 25 µF, 200 Ω using a Bio-Rad MicroPulser (California, USA). Immediately afterward, 1 mL of pre-warmed LB was added; cells were transferred to a 15 mL tube and recovered at 37°C with shaking (200 rpm) for 1 h to allow expression of antibiotic-resistance markers. Serial dilutions were plated on TSA supplemented with 10 µg/mL rifampicin and 0.02 µg/mL imipenem and incubated at 37°C for 18–24 h. Transformants were enumerated, and plasmid uptake was verified by PCR and antibiotic-susceptibility testing.

Electroporation was performed with cells maintained on ice (4°C) before and after the pulse, delivered at 2.5 kV, 25 µF, and 200 Ω. Recovery proceeded for 1 h at 37°C with shaking in 1 mL LB.

### Quantitative real-time PCR analysis of *acrAB-tolC* expression in *E. cloacae*

Total RNA was extracted from clinical isolates x9, F12 and x230151, together with the reference strain *E. cloacae* ATCC 13047, using a commercial kit (Tiangen Biotech, China). After DNase I treatment, cDNA was synthesised with gene-specific primers (**Supplementary Table S3**) and used to quantify the transcript levels of the *acrAB-tolC* efflux pump.

Expression of each target gene in clinical isolates x9, F12, and x230151, together with the reference strain ATCC 13047, was first normalized to the housekeeping gene *rpsL* to yield individual ΔCT values. These ΔCT values were then compared with the corresponding ΔCT of ATCC 13047 by calculating ΔΔCT (ΔCt, isolate - ΔCT, ATCC 13047). To standardize the assay, ATCC 27853 served as a positive calibrator: its ΔCT for each gene was set to 1 (**Table 3**), and the ΔCT values for isolates x9, F12 and x230151 were correspondingly rescaled (**Table 3**) before comparison with ATCC 13047. The final expression level reported for each isolate was the mean ΔΔCT derived from at least three independent biological replicates. Relative expression was classified according to the following thresholds: ΔΔCT > 0, denoted up-regulation (expression); ΔΔCT > 5, signified marked over-expression; and ΔΔCT < 0, indicated down-regulation (inactivation) or gene silencing.

**Table 3:** Gene expression of the *acrAB-tolC* from *E. cloacae* isolates x9, F12 and x230151.

RND Efflux pump gene	ΔCT value				ΔΔCT value			Interpretation of the result		
	ATCC 13047	x9	F12	x230151	x9	F12	x230151	x9	F12	x230151
*acrAB-tolC*	1	74.03	131.93	83.54	73.03	130.93	82.54	Over-expression	Over-expression	Over-expression
* acrA*	1	63.18	112.23	66.43	62.18	111.23	65.43	Over- expression	Over- expression	Over- expression
* acrB*	1	93.84	183.36	115.16	92.84	182.36	114.16	Over- expression	Over- expression	Over- expression
* tolC*	1	65.07	100.20	69.03	64.07	99.20	68.03	Over- expression	Over- expression	Over- expression

### Sequencing of the 16 S rRNA gene

Taxonomic identity was verified by amplifying the near-complete 16 S rRNA gene with the universal bacterial primers 27F (5′-AGAGTTTGATCMTGGCTCAG-3′) and 1492R (5′-GGTTACCTTGTTACGACTT-3′), where M denotes A or C. The reaction generated a single, robust amplicon of ~1.5 kb spanning the entire 16 S rRNA locus. Amplification was carried out in 50 µL volumes containing 1 × standard Taq buffer, 2.5 mM MgCl_2_, 0.2 mM dNTPs, 0.4 µM each primer, 2 U of a 3:1 mixture of Fermentas Taq DNA polymerase and Pfu high-fidelity enzyme (Thermo Fisher Scientific), and ~50 ng template DNA. Cycling parameters were: initial denaturation at 94°C for 3 min; 30 cycles of 94°C for 40 s, 50°C for 40 s, and 72°C for 1 min; and a final extension at 72°C for 5 min. Amplicons were purified and subjected to bidirectional Sanger sequencing to ensure high accuracy.

### Bacterial genome sequencing and *de-novo* assembly

We determined the complete genome sequences of *E. cloacae* clinical isolates x9, F12 and x230151 by integrating Oxford Nanopore and Illumina NovaSeq 6000 data. High-molecular-weight genomic DNA was mechanically sheared to an average fragment size of ~150 kb and ligated into Nanopore- compatible adapters for long-read sequencing, while a paired-end Illumina library with ~400 bp inserts was prepared in parallel to supply high-quality short reads. Raw Nanopore reads were first filtered to remove residual adapters and then error-corrected using *Canu v2.2* (https://github.com/marbl/canu), which trimed low-quality ends and applied a hierarchical assembly strategy to generate consensus sequences with reduced noise. Illumina reads were processed with *Trimmomatic v0.39* (https://github.com/usadellab/Trimmomatic) to clip Illumina adapters, discard bases below a Phred quality of 20, and eliminated any resulting reads shorter than 36 bp. After these quality-control steps, ~70.5 million Illumina reads remained, with an average length of 148 bp and 97.8% of bases scoring Q20 or higher. The combined data set provided more than 100-fold depth-of-coverage across the entire genome, ensuring complete representation of all replicons prior to assembly.

*De-novo* genome reconstruction was carried out using two complementary approaches. In the primary pipeline, we performed a hybrid assembly with *Unicycler v0.4.5* (https://github.com/rrwick/Unicycler), which first constructed an optimized de Bruijn graph from the Illumina reads via SPAdes, then resolved repetitive regions and joins contigs into complete replicons using the long Nanopore reads as scaffolds. A secondary Illumina-only assembly was generated with *SPAdes v3.15.3* (https://github.com/ablab/spades) using k-mer lengths ranging from 21 to 127 to provide an independent reference for gap filling and validation. Both assemblies were iteratively polished with *Pilon v1.24* (https://github.com/broadinstitute/pilon), which aligned the high-accuracy Illumina reads back to the draft contigs to correct single-nucleotide polymorphisms, small insertions/deletions, and local misassemblies, thereby maximizing base-level accuracy.

Following assembly, contigs were partitioned according to size and genomic context. The single contig exceeding 5 Mb was identified as the bacterial chromosome, consistent with the expected genome size of *E. cloacae*. All remaining contigs were provisionally classified as plasmids or plasmid fragments based on their smaller size, elevated read coverage, and the presence of plasmid-associated genes; this initial designation was subsequently confirmed through comprehensive gene annotation and comparative plasmid analysis.

### Comprehensive genome annotation and comparative genomics

Protein-coding potential was systematically delineated with *Prokka v1.14.6* (Seemann, 2014), leveraging Prodigal-driven gene prediction against curated databases to annotate intact open reading frames (ORFs) and flag disrupted pseudogenes. Functional curation followed a multi-tier pipeline: acquired resistance determinants were mined with *CARD 2023* (Alcock et al., 2023) and *ResFinder 4.0* (Bortolaia et al., 2020); insertion sequences were classified via IS*finder* (Siguier et al., 2006); integron structures were resolved with *INTEGRALL* (Moura et al., 2009); and transposon architecture was defined through the *Tn Number Registry* (Tansirichaiya et al., 2019). Multilocus sequence types (MLST) were assigned in silico by *MLST 2.0* (https://cge.food.dtu.dk/services/MLST/) and corroborated with *BacWGSTdb 2.0* (Feng et al., 2021).

For the plasmids carried by isolates x9, F12 and x230151, a full comparative workflow was executed. Complete plasmid backbones were first rendered as interactive, GC-skewed circular diagrams using *CGView v2.0.3* (Stothard et al., 2019). Pairwise and multiple alignments were performed with *BLAST v2.13.0^+^* (BLAST: Basic Local Alignment Search Tool); regions ≥ 90% nucleotide identity were highlighted as conserved syntenic blocks. The taxonomic relatedness among three ECC isolates (x9, F12, and x230151) was inferred from pairwise Average Nucleotide Identity (ANI) values calculated over their complete chromosome sequences using *JSpeciesWS* (https://jspecies.ribohost.com/jspeciesws/). Comparative diagrams were produced with the R package *genoPlotR v0.8.11* (http://genoplotr.r-forge.r-project.org/) and subsequently polished in *Inkscape v0.48.1* (https://inkscape.org/en) to deliver publication-ready vector graphics with uniform colour schemes, scalable legends and consistent resolution.

### Nucleotide sequence accession numbers

The complete nucleotide sequences of isolates x9, F12 and x230151 had been deposited in the NCBI GenBank database under the accession numbers listed in **Table 4**.

**Table 4:** Characterization of the *E. cloacae* DNA sequences harboring the NDM gene.

DNA Sequence	Plasmid Type	Length (kb)	GC%	Status	Antibiotic resistance gene	GenBank Accession no.
F12 strain						
F12_chr	/	4926.267	55.25	Complete	*bla*_ACT-17_/*fosA2/oqxA/acrAB-tolC*, etc^##^	CP199525.1
F12_p1	-^#^	118.568	52.03	Complete	*bla*_LAP-2_/*qnrS1*	CP199526.1
F12_p2	IncFII_(Yp)_	96.842	53.79	Complete	*bla*_TEM-1_/*qacEdelta1*/*sul1*/*bla*_NDM-1_/ *ble*_MBL_/*armA*/*msrE*/*mphE*	CP199530.1
F12_p3	IncR	81.859	52.81	Complete	*mphE*/*msrE*/*armA*/*sul1*/*qacEdelta1*/ *aadA1*/*bla*_TEM-1_/*bla*_SFO-1_/*aac(3)-IId*/ *catB3*/*bla*_OXA-1_/*aac(6')-Ib10*/*aph(3')-Ia*	CP199529.1
F12_p4	–	71.787	55.23	Complete	None	CP199528.1
F12_p5	Col_(pHAD28)_	6.211	52.52	Complete	*aph(3'')-Ib*/*aph(6)-Id*/*sul2*	CP199527.1
x230151 strain						
X230151_chr	/	4755.242	55.05	Complete	*bla*_ACT-24_/*fosA2/oqxAB/acrAB-tolC*, etc^##^	CP199512.1
X230151_p1	IncFII_(pECLA)_- *repE*_(pEh60-7)_	136.584	52.75	Complete	*bla*_TEM-1_(2)/*mrx*/*mphA*/*bla*_CTX-M-3_	CP199513.1
X230151_p2	IncFIB_(pHCM2)_	134.108	52.15	Complete	*qnrA1*/*sul1*/*bla*_NDM-1_/*ble*_MBL_/ *sul1*/*qacEdelta1*/*dfrA12*	CP199514.1
x9 strain						
x9_chr	/	4761.835	55.55	Complete	*bla*_ACT-17_/*fosA2/oqxA/acrAB-tolC*, etc^##^	CP199505.1
x9_p1	IncR-IncX3	106.063	52.89	Complete	*bla*_SHV-12_/*dfrA12*/*aadA2*/*qacEdelta1*/ *sul1*/*mrx*/*mphA*/*ble*_MBL_/*bla*_NDM-1_	CP199506.1
x9_p2	–	5.732	48.39	Complete	None	CP199507.1
x9_p3	Col_(pHAD28)_	2.699	50.39	Complete	*qnrB19*	CP199508.1
x9_p4	Col(_pHAD28)_	2.496	51.52	Complete	None	CP199509.1

^#^, no data; ^##^, for more detailed data, please refer to **Table S2**.

## Results

### Antimicrobial susceptibility testing, enzymatic characterization, transferable properties and pairwise ANI analysis among isolates x9, F12, and x230151

Isolates x9, F12 and x230151 were unambiguously confirmed as *E. cloacae* by high-confidence BLAST alignments of full-length 16 S rRNA genes (>99% identity) and by whole-genome phylogenomic analysis against the ECC isolates; pairwise ANI values of 98.79% (F12 vs x9), 95.42% (F12 vs x230151) and 95.36% (x9 vs x230151) all exceeded the 95% species threshold, further confirming that the three ECC isolates were conspecific. MLST resolved the isolates into ST116, ST93 and ST78 (**Table 1**), respectively. Antimicrobial-susceptibility testing revealed an extensive, clinically alarming resistance profile across all three strains, with carbapenem non-susceptibility as the defining phenotype (MIC ≥ 8 mg/L for imipenem, meropenem, ertapenem and doripenem) (**Table 2**). This multidrug- resistant signature was underpinned by a layered resistance network: chromosomally encoded *bla*_ACT_-type AmpC β-lactamases and an over-expressed *acrAB-tolC* efflux system, together with plasmid-borne *bla*_NDM-1_ carbapenemase (**Tables 2**, **4**), *bla*_TEM-1_ and *bla*_LAP-2_ or *bla*_TEM-1_ and *bla*_CTX-M-3_*or bla*_SHV-12_ class A β-lactamases (**Table 4**), and aminoglycoside-modifying *armA* and *aadA1*, etc or *aadA2* (**Table 2**). Bacterial conjugation and electroporation successfully recovered the integrated plasmids at the following average transfer frequencies: F12_p2 and F12_p3 at 4.48×10–^3^ and 0.971×10^-3^, x130151_p1 and x130151_p2 at 8.06×10–^3^ and 3.84×10^-3^, and x9_p1 at 5.05×10^-3^ (**Supplementary Table S1**).

### Gene expression of the *AcrAB-TolC* efflux system in isolates x9, F12, and x230151

RNA-seq analysis demonstrated concerted over-expression of chromosomal *acrA*, *acrB*, and *tolC* in isolates x9, F12, and x230151 (**Table 3**), leading to marked over-expression of the entire AcrAB-TolC efflux system (**Table 3**). This coordinated transcriptional activation resulted in a significant upregulation of the entire efflux system, demonstrating that the multidrug-resistant phenotype observed in routine clinical susceptibility testing was attributable (**Table 2**), at least in part, to enhance antibiotic efflux mediated by the AcrAB-TolC pump - a mechanism affecting agents such as fluoroquinolones, β-lactams chloramphenicol, tetracyclines, and glycylcycline (**Table 2**).

### Characterization of drug-resistance genes on chromosome of isolates x9, F12, and x230151

Genomic dissection of isolates x9, F12, and x230151 revealed distinct yet uniformly complex architectures. x9 comprised a single chromosome plus four plasmids, F12 harbored a chromosome and five plasmids, and x230151 carried a chromosome and two plasmids (**Table 4**). In-depth annotation identified 33, 35, and 33 chromosomally encoded drug-resistance determinants or cognate operons in x9, F12, and x230151, respectively (**Supplementary Table S2**), underscoring a conserved, high-density resistance repertoire. Most conspicuous were the RND-type AcrAB-TolC efflux pump and the class C β-lactamase gene *bla*_ACT_, both ubiquitously presented across the three isolates, serving as pivotal drivers of antibiotic resistance. Although the three isolates acquired markedly different chromosomal architectures, they converged on a shared phenotype of broad-spectrum antibiotic resistance (**Table 2**). Two mutually reinforcing forces propelled the evolutionary convergence. On the one hand, the constitutive hyper- production of the AcrAB-TolC efflux system operated in tandem with *bla*_ACT_-driven β-lactam hydrolysis to establish a robust, intrinsic resistance platform. On the other, this genetic scaffold was amplified by an ensemble of synergistic, plasmid-encoded factors, foremost among them the *bla*_NDM-1_ carbapenemase, that further elevated the resistance phenotype. Together, these factors severely compromised the efficacy of antimicrobial therapy.

### Comprehensive comparative analyses of the intact F12_p3 plasmid against plasmids, pEF7-18-58 _2 (CP068593.1), pKP110-1 (CP144127.1), and pKSC24 (KX443408.2)

The complete F12_p3 plasmid carried a repertoire of thirteen discrete antibiotic-resistance determinants: the β-lactamases *bla*_TEM-1_, *bla*_SFO-1_, and *bla*_OXA-1_ (a class D oxacillinase), the aminoglycoside modifiers *aph(3′)-Ia*, *armA*, *aac(3)-IId*, *aadA1*, and *aac(6′)-Ib10*, the macrolide- resistance module *mphE*-*msrE*, the chloramphenicol acetyltransferase *catB3*, the sulfonamide-resistance gene *sul1*; and the quaternary-ammonium compound efflux determinant *qacEdelta1* (**Tables 2**, **4**; **Supplementary Figure S1**), and the genes *intI1*, *aadA1*, *qacEdelta1*, and *sul1* collectively defined the intact class 1 integron In2. To precisely map the distribution of specific resistance determinants, we performed an exhaustive, nucleotide-resolution comparison between the intact F12_p3 plasmid and its closest relatives, pEF7-18-58_2, pKP110-1, and pKSC24, focusing on both antibiotic-resistance gene inventory and global structural homology. Pairwise alignments and whole-plasmid synteny analyses revealed negligible collinearity among these replicons; more importantly, their resistance-gene repertoires diverged markedly. The unique resistance modules harbored by F12_p3 were either entirely absent or rearranged beyond recognition in the comparator plasmids (**Supplementary Figure S1**). Collectively, these findings highlighted a profound, genome - wide structural discordance that extended from the core backbone to the accessory antibiotic-resistance elements.

### Comprehensive comparative analyses of plasmid x230151_p1 with the complete sequences of cre46 plasmid unnamed1 (CP039385.1), p44-1 (CP025462.1), and pA1718-HI3 (MW013142.1)

The complete x230151_p1 carried a repertoire of five discrete antibiotic-resistance determinants: the β-lactamases 2 *bla*_TEM-1_, and *bla*_CTX-M-3_; a functionally coupled macrolide-resistance module *mphA*- *mrx* (**Tables 2** and **4**, **Supplementary Figure S2**). Pairwise alignment showed that > 66% of the complete x230151_p1 sequence aligned end-to-end with cre46 plasmid unnamed1, with the single exception of the *bla*_CTX-M-3_- bearing segment (**Supplementary Figure S2**). In stark contrast, only short, patchy homologies, restricted to the duplicated *bla*_TEM-1_ loci plus the *mphA*-*mrx* module, were shared with p44–1 or pA1718-HI3; notably, no *bla*_CTX-M-3_ regions were also found in either of the latter two replicons (**Supplementary Figure S2**).

### Comprehensive comparative analyses of x9_p1 with plasmids pCRE40_1 (CP074559.1), pSL131_ IncA/C-IncX3 (MH105050.1) and the chromosome of strain 65 (CP128442.1)

**Figure 1:**
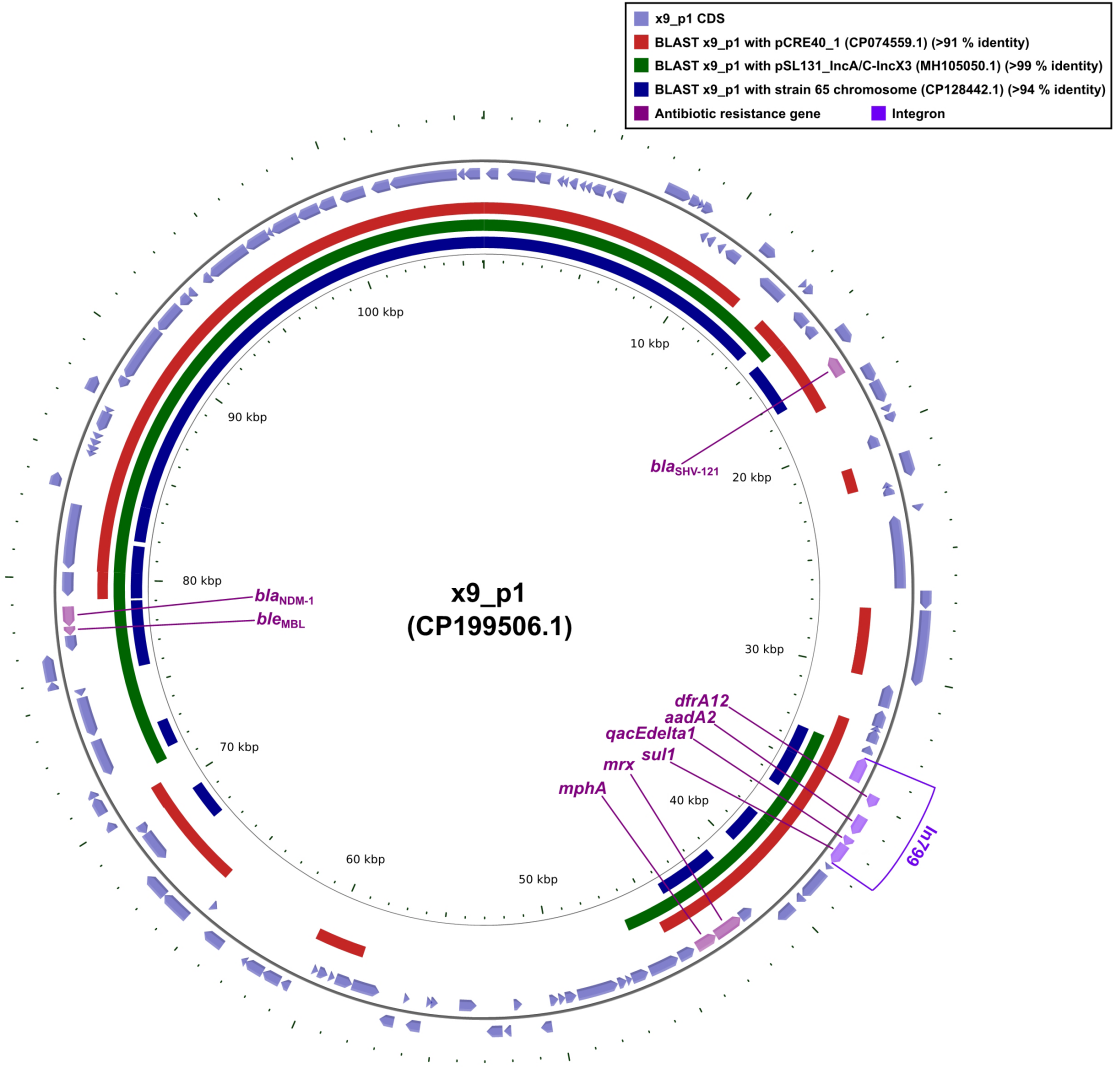
Comparative plots of the complete x9_p1 plasmid against pCRE40_1, pSL131_IncA/C-IncX3, and the partial chromosome of strain 65. The figure was produced with the R package *genoPlotR v0.8.11*, and illustrated manually using *CGView v2.0.3*. The locations of the antibiotic resistance genes were using *Inkscape 0.48.1* (https://inkscape.org/en). High-identity alignment data for plasmid x9_p1 versus pCRE40_1, pSL131_IncA/C-IncX3, and the partial chromosome of strain 65 were provided in **Supplementary Materials 1-3**.

Plasmid x9_p1 harbored nine distinct resistance determinants: the β-lactamases *bla*_SHV-121_ and *bla*_NDM-1_ (a class B carbapenemase), the bleomycin-resistance gene *ble*_MBL_, which was tightly linked to *bla*_NDM-1_, the macrolide-resistance module *mphA-mrx*, the dihydrofolate reductase *dfrA12*, the aminoglycoside-modifying *aadA1*, the sulfonamide-resistance gene *sul1*, and the quaternary- ammonium efflux pump *qacEdelta1* (**Tables 2** and **4**, **Figure 1**). Within this repertoire, *intI1*, *dfrA2*, *aadA1*, *qacEdelta1*, and *sul1* together constituted the class 1 integron In799.

Sequence comparisons revealed that plasmid pCRE40_1 aligned with x9_p1 over ≥ 50% of its length but lacked the blaNDM-1-bleMBL cassette (**Figure 1**), indicating that these replicons were already partially homologous before the resistance unit was acquired. In contrast, plasmid pSL131_IncA/C -IncX3 and the chromosome of strain 65 shared ≥ 50% identity and both harbored *bla*_NDM-1_-*ble*_MBL_ (**Figure 1**), implying that the same module had been independently inserted into distinct plasmid and chromosomal loci. Taken together, the evidence indicated that the *bla*_NDM-1_-*ble*_MBL_ module was not an integral part of the pCRE40_1 or pSL131_IncA/C-IncX3 backbones; instead, it was carried by a discrete mobile unit, most likely a composite transposon typified by Tn*125* or IS*Aba125*, that could shuttle laterally among IncA/C, IncX3 and chromosomal scaffolds while maintaining genetic coherence even in markedly dissimilar hosts.

### Comprehensive comparative analyses of plasmid F12_p2 against pEA49-KPC (KU318419.1), p72_4 (CP101558.1), and F5111 (KU987453.1)

**Figure 2:**
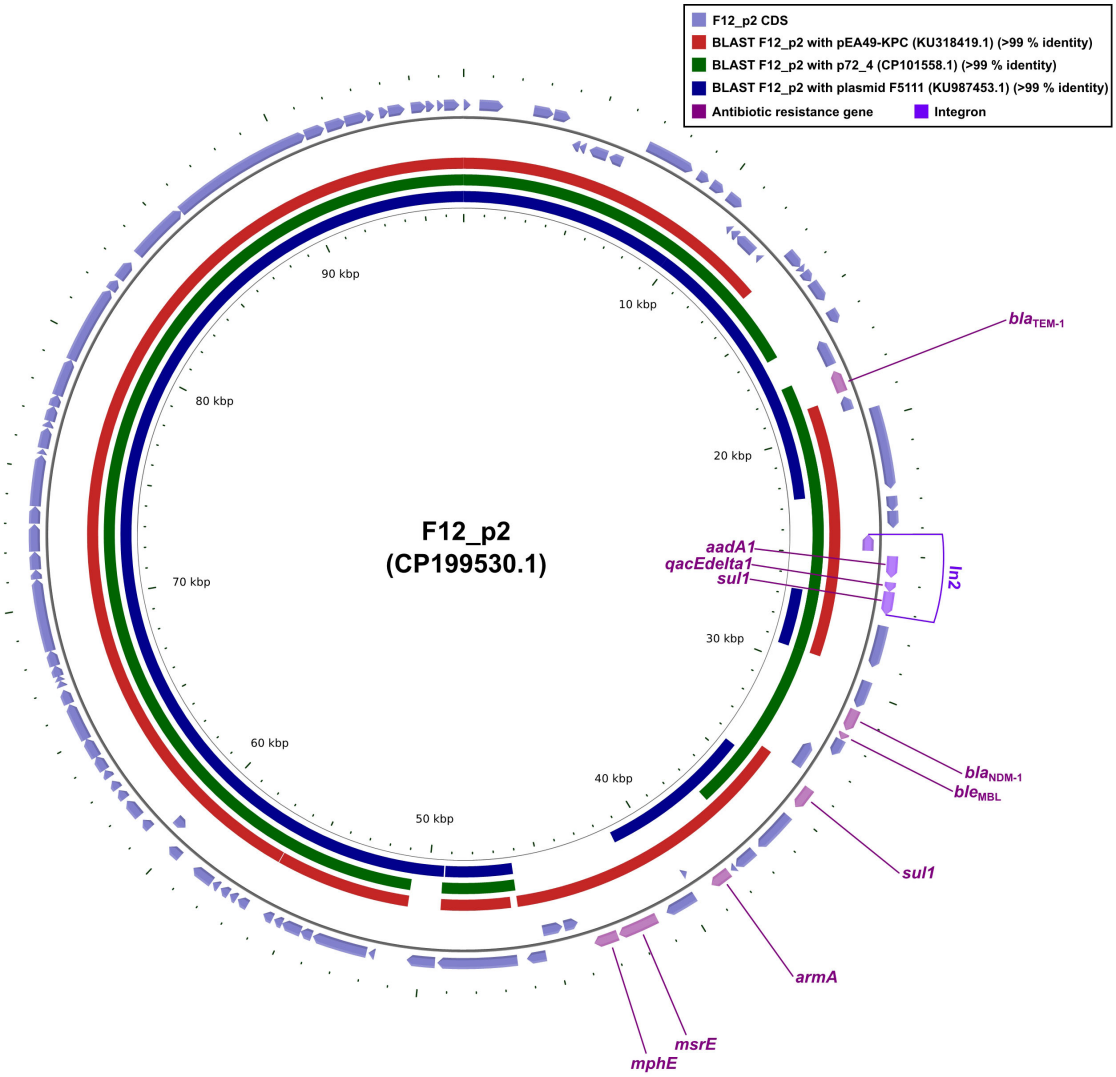
Whole-plasmid comparisons of F12_p2 with pEA49-KPC, p72_4 and F5111. Comparative diagrams were produced with the R package *genoPlotR v0.8.11*, and illustrated manually using *CGView v2.0.3*. Antibiotic-resistance loci were manually annotated with *Inkscape 0.48.1* (https://inkscape.org). High-identity alignments were provided in **Supplementary Materials 4-6**.

Plasmid F12_p2 carried ten resistance determinants: the β-lactamases *bla*_TEM-1_ and *bla*_NDM-1_ (a class B carbapenemase); the bleomycin-resistance gene *ble*_MBL_, tightly linked to *bla*_NDM-1_; the aminoglycoside- modifying enzyme *aadA1* and the 16S rRNA methyltransferase *armA*; two copies of the sulfonamide- resistance gene *sul1*; the quaternary-ammonium efflux pump *qacEdelta1*; and the macrolide-resistance module *mphE-msrE* (**Figure 2**). Within this array, *intI1*, *aadA1*, *qacEdelta1*, and the immediately adjacent *sul1* together constitute the class 1 integron In799 (**Figure 2**).

Sequence alignments showed that plasmids pEA49-KPC, p72_4 and F5111 each matched ≥90% of the F12_p2 core backbone (**Figure 2**). Yet p72_4 and F5111 lacked the *bla*_NDM-1_-*ble*_MBL_ module, suggesting these replicons were already highly homologous before acquisition of the resistance unit. Conversely, pEA49-KPC shared ≥90% identity with F12_p2 and carried the same *bla*_NDM-1_-*ble*_MBL_ (**Figure 2**). These data revealed that pEA49-KPC and F12_p2 shared virtually identical core backbones long before the *bla*_NDM-1_-*ble*_MBL_ module appeared. Subsequently, the resistance locus was either captured once and then propagated vertically, or implanted independently, yet identically, by a shared mobile element that mobilized after plasmid divergence or while the plasmids still co-resided in the same cytoplasm. When considered together, the data cast *bla*_NDM-1_-*ble*_MBL_ as an extrinsic, ultra-mobilizable module that could glide between highly congruent replicons, preserving both the host architecture and its own genetic coherence.

### Comprehensive comparative analyses of x230151_p1 with plasmids CRECL11 plasmid unnamed1 (CP166889.1), pECC-143-1 (CP143709.1) and pECC-102-1 (CP143730.1)

**Figure 3:**
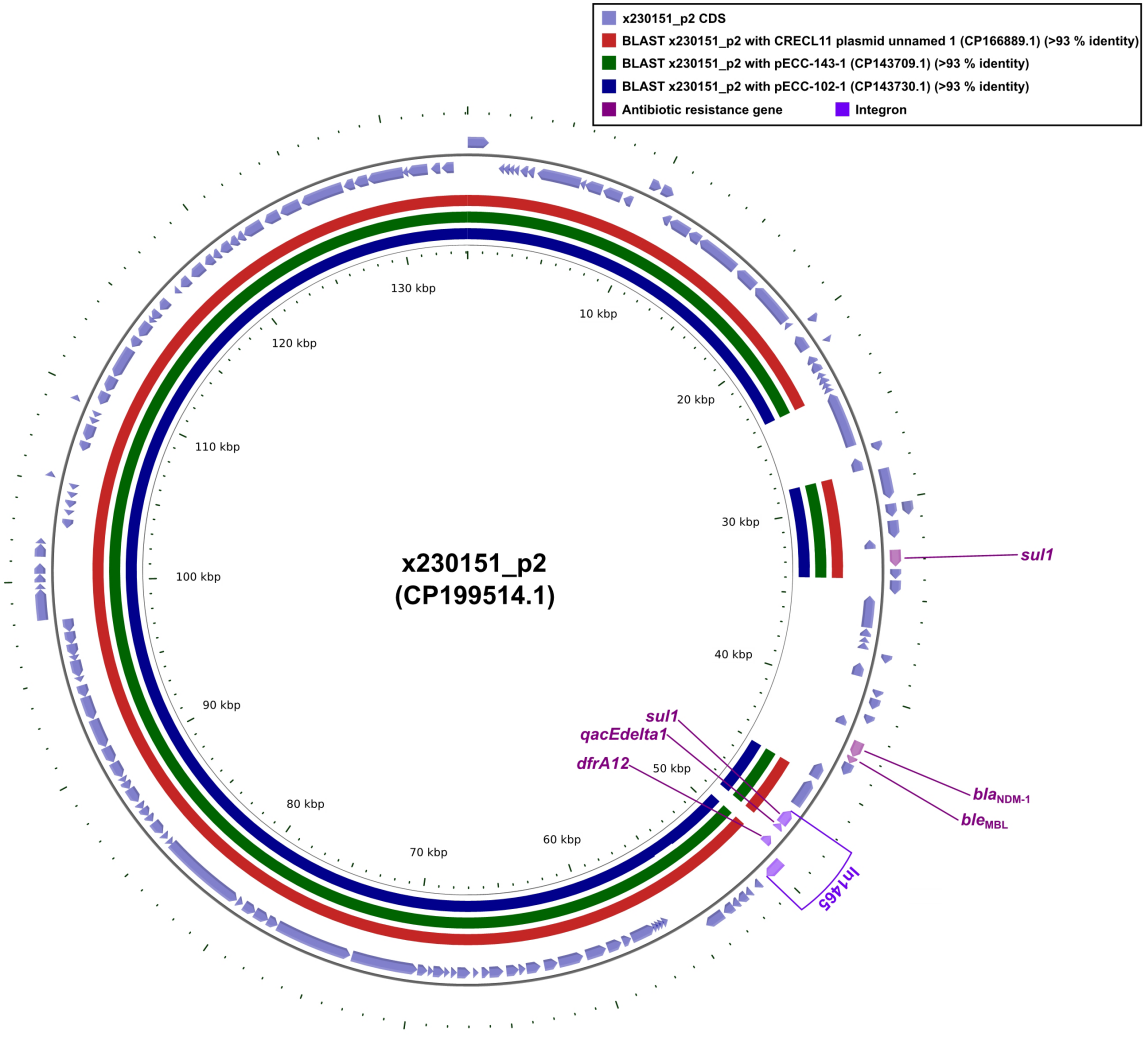
Comparative plots of the complete x230151_p2 plasmid against CRECL11 plasmid unnamed 1, pECC-143-1, and pECC-102-1. The figure was produced with the R package *genoPlotR v0.8.11*, and illustrated manually using *CGView v2.0.3*. The locations of the antibiotic resistance genes were illustrated manually using *Inkscape 0.48.1* (https://inkscape.org/en). High-identity alignment data for plasmid x230151_p2 versus CRECL11 plasmid unnamed 1, pECC-143-1, and pECC-102–1 were provided in **Supplementary Materials 7-9**.

Plasmid x230151_p1 encoded a compact but potent resistance cluster of six genes. Chief among them was *bla*_NDM-1_, a class B metallo-β-lactamase that hydrolysed carbapenems, physically linked to its bleomycin-shielding partner, *ble*_MBL_. Downstream lied the dihydrofolate reductase *dfrA12*, two copies of the sulfonamide-resistance determinant *sul1*, and the quaternary-ammonium efflux pump *qacEdelta1* (**Figure 3**). These four loci, *intI1*, *dfrA12*, *qacEdelta1*, and the adjacent *sul1*, coalesced into the class 1 integron In1465, a mobile genetic platform that disseminated multidrug resistance across diverse bacterial hosts (**Figure 3**).

Whole-plasmid alignments revealed that CRECL11 plasmid unnamed1, pECC-143-1, and pECC-102–1 each shared ≥90% nucleotide identity across the x230151_p1 core backbone (**Figure 3**). Strikingly, all three lacked the contiguous *bla*_NDM-1_-*ble*_MBL_ module. These findings established that the *bla*_NDM-1_-*ble*_MBL_ module was neither ancestral nor integral to the core replicons examined. Rather, it represented a laterally acquired, self-contained resistance module that was inserted independently into an otherwise highly conserved plasmid scaffold. Before this insertion, CRECL11 plasmid unnamed 1, pECC-143-1, and pECC-102–1 already shared ≥90% identity with x230151_p1 across their backbones, implying descent from a common, stable progenitor. The subsequent, sporadic appearance of *bla*_NDM-1_- *ble*_MBL_ in only one lineage underscored the mobility of the unit, most likely mobilized by composite transposons or integrons, and its ability to vault between closely related yet distinct plasmid backgrounds without perturbing the underlying architecture.

### Comprehensive comparative analyses on the *bla*_NDM-1_-*ble*_MBL_-carrying plasmids x9_p1, F12_p2, and x230151_p1, using the reference plasmids F11_plasmid C (CP092904.1) or F11_plasmid B (CP092903.1) as benchmarks

**Figure 4:**
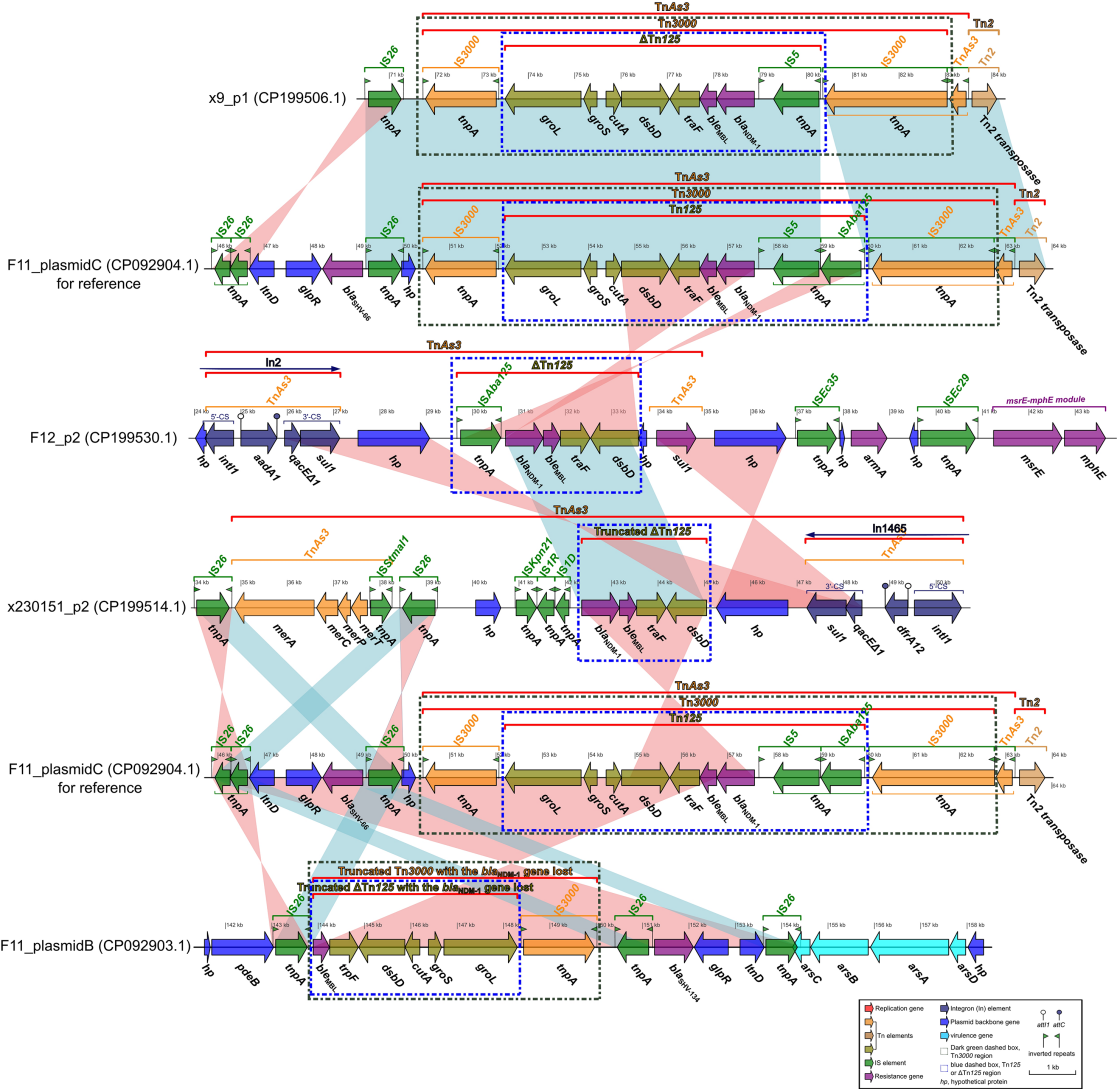
Comparative analysis of the *bla*_NDM-1_-*ble*_MBL_-harboring plasmids x9_p1, F12_p2, and x230151_p1, benchmarking each against reference sequences F11_plasmid C or F11_plasmid B. Comparative diagrams were first generated with the R package *genoPlotR v0.8.11* and subsequently refined and annotated using Inkscape 0.48.1 (https://inkscape.org).

F11_plasmid C and F11_plasmid B were two high-molecular-weight plasmids that were isolated from the clinical *K. pneumoniae* strain F11 recovered at our hospital (Ma et al., 2024). F11_plasmid C harbored the carbapenem-resistance determinant *bla*_NDM-1_ together with its cognate bleomycin-resistance gene *ble*_MBL_, forming a contiguous and structurally conserved *bla*_NDM-1_-*ble*_MBL_ resistance module (**Figure 4**). In F11_plasmid B, the paradoxical loss of the core *bla*_NDM-1_ gene alongside the preservation of an otherwise intact *bla*_NDM-1_-*ble*_MBL_ resistance module was striking. The most plausible explanation was a precisely executed, site-specific recombination event that surgically removed the carbapenemase determinant. This observation not only underscored the extraordinary structural stability of the *bla*_NDM-1_- *ble*_MBL_ core backbone, but also revealed a cunning bacterial evolutionary strategy: by reversibly shedding a costly resistance gene, the pathogen could swiftly adapt to fluctuating antibiotic pressures while maintaining a genomic”docking site”that allowed for rapid re-acquisition (**Figure 4**). These findings carry profound implications for deciphering the molecular mechanisms underlying resistance dissemination and for refining clinical surveillance strategies.

Comparative analysis of plasmid x9_p1 and F11_plasmid C revealed that the *bla*_NDM-1_-*ble*_MBL_- bearing segment of x9_p1 (approximate 70–85 kb) was almost identical to the corresponding region of F11_plasmid C (approximate 49–64 kb). Both regions displayed complete or essentially complete Tn*3000* and Tn*125* architectures (**Figure 4**), with the exception that the IS5-downstream IS*3000* interval lacked IS*Aba125* in x9_p1. The canonical structures were as follows: Tn*3000*, IS*3000*-*groL-groS- cutA-dsbD-traF-ble*_MBL_-*bla*_NDM-1_-IS*5*-(IS*Aba125*)-IS*3000*; and Tn*125* (or ΔTn*125*), *groL-groS-cutA- dsbD-traF*-*ble*_MBL_-*bla*_NDM-1_-IS*5*-(IS*Aba125*).

Alignment of F12_p2 with F11_plasmid C showed that the *bla*_NDM-1_-*ble*_MBL_ segment of F12_p2 (approximate 29.5-33.5 kb) matched the corresponding region of F11_plasmid C in reverse orientation, comprising only a truncated ΔTn*125*, lacking the full Tn*3000* structure, with the arrangement “IS*Aba125*-*bla*_NDM-1_-*ble*_MBL_-*traF*-*dsbD*” (**Figure 4**). Plasmid x230151_p2 carried an even more truncated ΔTn*125* fragment containing *bla*_NDM-1_-*ble*_MBL_-*traF*-*dsbD* (**Figure 4**). Comparison between F11 plasmids C and B revealed that plasmid B contained two inverted, *bla*_NDM-1_-deficient remnants: a truncated Tn*3000* retaining *ble*_MBL_-*traF-dsbD-cutA-groS-groL*-IS*3000* (**Figure 4**), and a shorter ΔTn*125* fragment that also lacked *bla*_NDM-1_ but involved the *ble*_MBL_-*traF-dsbD-cutA-groS-groL* gene cluster (**Figure 4**). Comparative plasmid genomics has revealed four interconnected lessons. (1) Backbone versus ornaments: a stable Tn*3000*/Tn*125* core backbone underpins every *bla*_NDM-1_-*ble*_MBL_ module examined, yet IS*Aba125*, IS*3000* and related insertion sequences behave like snap-on modules that are readily added, inverted or discarded during both conjugative transfer and intramolecular shuffling. (2) Recombination as a molecular workshop: Inverted blocks, pinpoint IS*Aba125* losses and sequential truncations all point to site-specific recombination, whether triggered by IS transposition, homologous exchange or XerC/D- tyrosine recombinases, as the dominant driver. The region operates as a high- throughput “cut-and-paste” facility that continually re-engineers the module. (3) A reversible gene- offload tactic: F11_plasmid B excises *bla*_NDM-1_ but retains the downstream gene cluster, leaving an empty docking site. Because the same cell still harbors F11_plasmid C with an intact *bla*_NDM-1_ copy, the strain can toggle carbapenem resistance on or off via inter-plasmid complementation or rapid re-acquisition - a reversible evolutionary switch. (4) Surveillance blind spots: PCR screens that merely ask “*bla*_NDM-1_ present/absent?” risk missing silent plasmids poised for re-activation. Incorporating structural-integrity assays and real-time IS dynamics will expose latent threats, while the unique recombination fingerprints generated in this hotspot can serve as new epidemiological markers.

## Discussion

This study dissected the twin engines driving multidrug resistance in prevalent ECC clinical isolates: constitutive over-expression of the AcrAB-TolC efflux pump and the archetypal carbapenemase *bla*_NDM-1_. MLST assigned ECC isolates (x9, F12 and x230151) to ST116, ST93 and ST78, respectively, with ST93 and ST78 emerging as high-risk clones linked to global nosocomial outbreaks (Cai et al., 2024). Further characterization revealed that all three isolates (x9 and x230151 and F12) co-produced β-lactamases of classes A, B and C, while isolate F12 whereas co-produced additionally a class D β-lactamase (**Table 1**). Collectively, these enzymes endowed the isolates with pan-β-lactam resistance, allowing them to fully evade carbapenem activity (**Table 2**). Conjugation frequencies of ~10–^3^ ensured plasmid persistence within parental cells and efficient horizontal dissemination, fueling outbreaks of carbapenem-resistant ECC that severely compromised therapeutic options.

The *acrAB-tolC* locus encoded a canonical RND-family efflux pump whose functional core comprised three tightly orchestrated components: the inner-membrane proton/drug antiporter AcrB, the periplasmic adaptor AcrA, and the trimeric outer-membrane channel TolC (Kim et al., 2015). Acting as a single, high-efficiency conduit that spanned both membranes and the intervening periplasm, this complex captured and expelled a structurally diverse array of antimicrobials, including fluoroquinolones, chloramphenicol, tetracyclines, macrolides, and others, before they could reach intracellular targets (Jang, 2023). The net effect was a sharp reduction in intracellular drug accumulation and a concomitant rise in MICs across multiple antibiotic classes (Jang, 2023). Transcriptional control of the operon was primarily governed by the AraC/XylS-type activator RamA (Kim et al., 2015). Once induced, RamA engaged specific binding motifs within the *acrAB* and *tolC* promoters, triggering a transcriptional burst that doubles efflux capacity within a single bacterial generation (Kim et al., 2015).

*AcrAB-tolC* overexpression operated on two distinct but synergistic levels: immediate efflux-mediated drug tolerance and long-term mutator-driven evolution. Effective therapeutic strategies must therefore target both the pump itself and the regulatory circuits that sustain its expression and evolutionary plasticity. (1) It directly caused overproduction of the AcrAB-TolC efflux pump, leading to marked increases in MICs for multiple drug classes, including fluoroquinolones, chloramphenicol, tetracyclines, and macrolides, and thus an MDR phenotype (**Tables 2**, **3**). (2) Indirectly, it established a mutator state: *acrAB-tolC* overexpression down-regulated the DNA mismatch-repair gene *mutS*, elevating the global mutation rate and accelerating the accumulation of high-level resistance mutations such as *gyrA* fluoroquinolone-target alterations (**Supplementary Table S2**) (Grimsey et al., 2020); additionally, compensatory mutations could arise: when the RamA pathway was already maximally induced (e.g., in *ramR*-deletion backgrounds), bacteria might acquire mutations in the SoxRS system, such as point mutations in *soxR* (**Supplementary Table S2**), that further up-regulated *acrAB* expression, preserving a selective advantage. This underscored that single-enzyme inhibitors might be insufficient to reverse the resistance (Grimsey et al., 2020).

The *bla*_NDM-1_ gene encoded a metallo-β-lactamase that coordinated two Zn^2+^ ions in its active site and hydrolyzed nearly every β-lactam antibiotic, penicillins, cephalosporins, and carbapenems alike. Notably, it only achieved full resistance to monobactams such as aztreonam when cooperating with additional enzymes (Melgarejo et al., 2019). Carried by conjugative plasmids (**Supplementary Table S1**) or integrons, and Transposon (**Figure 4**), *bla*_NDM-1_ was driven by potent upstream promoters, IS*Aba125* among them, that ensured robust transcription. Elevated enzyme titers could raise imipenem and meropenem MICs 8- to 64-fold (Melgarejo et al., 2019). When combined with porin loss (e.g., ompK36) or over-expression of the AcrAB-TolC efflux pump, the phenotype escalated to multi-drug resistance (**Table 2**). Transient transcriptional or translational boosted in either *acrAB-tolC* or *bla*_NDM-1_ could instantaneously increase MICs, while sustained AcrAB-TolC over-expression fostered a mutator phenotype that accelerated secondary mutations in target genes, regulatory circuits, and even the β-lactamase locus itself, driving stepwise resistance evolution.

Therapeutically, single-agent inhibition of RamA or NDM-1 risked activating compensatory networks, e.g., *soxRS*, and AmpC (**Supplementary Table S2**). Consequently, combination regimens pairing efflux inhibitors, β-lactamase inhibitors, and porin-enhancing agents were required to curb the emergence of resistance. Despite their potent inhibition of the AcrAB-TolC efflux pump *in vitro* and in murine models, phenylalanine-arginine-β-naphthylamide (PAβN) and the pyridopyrimidine lead D13–9001 had yet to advance into clinical development, hindered by toxicity, a narrow spectrum of activity, or unfavorable pharmacokinetics (Zhang et al., 2023). Accordingly, further research was needed to address these limitations and explored potential modifications or alternative compounds that could enhance their therapeutic efficacy while minimizing adverse effects. With all three isolates (F12, x9, and x230151) simultaneously over-expressing a hyper-active AcrAB-TolC efflux pump and NDM-1, clinicians were left with only one actionable target: the NDM-1 determinant itself. Yet evidence was unambiguous: carbapenem MICs above 4–8 mg/L rendered every β-lactam/β-lactamase-inhibitor combination ineffective (Hu et al., 2025), and high-level *bla*_NDM-1_ transcription could push MICs 8- to 64-fold higher (**Table 2**); once above 32 mg/L, neither extended nor continuous infusion offerred any therapeutic recourse (Sartor et al., 2014). Although salvage triple regimens, ceftazidime-avibactam plus aztreonam (when a metallo-β-lactamase was co-expressed), tigecycline, eravacycline, or polymyxins, had been advocated, however, none had translated into meaningful clinical benefit for these three isolates (F12, x9, and x230151) (**Table 2**). Accordingly, real-time genomic surveillance, tracking *ramR* and *soxR* mutations alongside insertions, deletions, or mobile elements such as IS*Aba125* within the *bla*_NDM-1_ promoter, should therefore serve as an early molecular sentinel to pre-empt the emergence of high-level resistance exemplified by isolates F12, x9, and x230151.

Furthermore, comparative plasmid analysis of isolates, F12, x9, and x230151 (**Figures 1**, **2**, **3**), revealed an apparent paradox: the *bla*_NDM-1_-*ble*_MBL_ module was both highly mobile and remarkably stable. This duality could be rationalised by four convergent mechanisms. (1) A lock-and-key transposon scaffold: nested within the classical composite transposon Tn*125*, the module was flanked by IS*Aba125* inverted repeats that retained functional transposase genes, enabling rapid excision and re-insertion (Weber et al., 2019). Simultaneously, the Tn*125* core was physically coupled to the partition systems (*parAB* and *stbAB*) of IncFII/IncN plasmids; these systems actively segregated plasmid copies during replication, effectively “locking” the module in place and minimising loss (Weber et al., 2019). (2) IS*26*-mediated second-order petrification: in German outbreak isolates, IS*Aba125* was frequently interrupted by IS*26*, generating an IS*26*-IS*Aba125*-*bl*a_NDM_-*ble*_MBL_-IS*26* composite. Although transposition competence was retained, the altered terminal repeats lowered transposition frequency by one to two orders of magnitude, yielding a “low-frequency yet mobilisable” phenotype (Weber et al., 2019). (3) Host-plasmid co-evolutionary pressure: downstream genes *ble*_MBL_ and *sul1* formed a co-selected resistance unit. Under the cumulative pressure of carbapenems and other antibiotics, the host’s reliance on the plasmid intensified, thereby firmly anchoring the resistance module; conversely, when antibiotic pressure was removed, IS*26*-mediated deletions could excise the entire module within 48 h, producing an environment-dependent stability (Zhou et al., 2022). (4) Region-specific insertion sequences and promoter locking: in East-Asian isolates, IS*5166* was present upstream of *bla*_NDM-1_ in ~30% of IncX3 plasmids. IS*5166* enhanced the *bla*_NDM-1_ promoter, increasing metabolic load and imposing negative feedback on transposase expression. The resulting high-expression/high-cost equilibrium stabilised the module within IncX3 backbones (Acman et al., 2022). Collectively, the mobility of the *bla*_NDM-1_-*ble*_MBL_ module was driven by IS*Aba125*/IS*26*-mediated transposition, while its stability was enforced by IncF/IncN/IncX3 scaffolds, partition systems, and host-plasmid co-evolution, together establishing an environment-dependent dynamic equilibrium.

Of course, the following limitations of this study should be acknowledged: (1) Small sample size: Only three ECC clinical isolates (x9, x230151, and F12) were subjected to in-depth analysis, limiting statistical power and the generalizability of conjugation-frequency estimates and resistance-gene prevalence data. (2) Single-centre and geographic restriction: All isolates were obtained from one tertiary-care hospital in Taizhou, China, and may not reflect the resistance landscape in other regions or countries. (3) Short sampling window: Strains were collected over a 6-month period in 2022, precluding assessment of seasonal or long-term trends in antimicrobial resistance. (4) Absence of clinical data: Patient treatment response, mortality, and length of stay were not documented, preventing correlation of the observed resistance mechanisms with therapeutic failure.

## Conclusion

In clinically dominant ECC, acrAB-tolC over-expression and *bla*_NDM-1_ functioned as a synergistic resistance axis. The efflux pump rapidly lowered intracellular antibiotic concentrations, while the metallo-β-lactamase hydrolyzed any residual β-lactam; together, they accelerated mutational escape. The *bla*_NDM-1_-*ble*_MBL_ module was both highly mobile, propelled by IS*Aba125*/IS*26* transposition, and paradoxically stable, anchored to IncF/IncN/IncX3 plasmids by robust partitioning systems and host-plasmid co-evolution under sustained antibiotic pressure. Each of the three isolates (x9, F12, and x230151) met XDR criteria, exhibiting carbapenem MICs of at least 8 mg/L. Consequently, containment required real-time genomic surveillance for *ramR*/*soxR* mutations and IS*Aba125*-driven promoter rearrangements, coupled with combination regimens that simultaneously suppressed efflux, β-lactamase activity, and plasmid persistence; strategies lacking any of these components only hastened the inevitable march toward XDR.

## Data Availability

The datasets presented in this study can be found in online repositories. The names of the repository/repositories and accession number(s) can be found in the article/**Supplementary Material**.

## Supplementary material

Supplementary Table 1 Profiles of bacterial conjugation and plasmid transfer via electroporation.

Supplementary Table 2 Characterization of chromosomally encoded drug-resistance genes in strains F12, x9 and x230151.

Supplementary Table 3 Primers for the *acrAB*-*tolC* gene in *Enterobacter cloacae.*

Supplementary Figure 1 Comparative plots of the complete F12_p3 plasmid against pEF7-18-58_2, pKP110-1, and pKSC24. The figure was produced with the R package *genoPlotR v0.8.11*, and illustrated manually using *CGView v2.0.3*. The locations of the antibiotic resistance genes were using *Inkscape 0.48.1* (https://inkscape.org/en). High-identity alignment data for plasmid F12_p3 versus pEF7-18-58_2, pKP110-1, and pKSC24 were provided in **Supplementary Materials (1-1) to (1-3)**.

Supplementary Figure 2 Comparative diagram of the complete x230151_p1 plasmid against cre46 plasmid unnamed 1, p44-1, and pA1718-HI3. The figure was produced with the R package *genoPlotR v0.8.11*, and illustrated manually using *CGView v2.0.3*. The locations of the antibiotic resistance genes were using *Inkscape 0.48.1* (https://inkscape.org/en). High-identity alignment data for plasmid x230151_p1 versus cre46 plasmid unnamed 1, p44-1, and pA1718-HI3 were provided in **Supplementary Materials (1-4) to (1-6)**.
