# Supplementary material for: Characterization of Enterobacter cloacae complex clinical isolates: comparative genomics and the role of the efflux pump AcrAB-TolC over-expression and NDM-1 production
Source: Front Cell Infect Microbiol. 2025 Nov 7;15:1705370. doi: 10.3389/fcimb.2025.1705370 (PMC12635725; doi:10.3389/fcimb.2025.1705370)
Supplement: Supplementary Table 1 — Profiles of bacterial conjugation and plasmid transfer via electroporation. [file Table1.docx]

| **Table S1** Profiles of bacterial conjugation and plasmid transfer via electroporation | | | | | | | | | | |
| --- | --- | --- | --- | --- | --- | --- | --- | --- | --- | --- |
| **Strain/plasmid** | | | **Bacterial conjugation and electroporation transfer** | | | | | | | |
|  |  |  | **Bacterial**  **conjugation** |  | **Conjugation frequency of plasmid electroporation transfer (×10^-3^)** | | | | | |
| **No. of experiments** | | | 5 |  | No. 1 | No. 2 | No. 3 | No. 4 | No. 5 | **Average** |
| **F12 strain** | |  | 4 successes  and 1 failure |  |  | | | | | |
|  | F12_p2 | |  |  | 4.16 | 3.81 | 5.05 | 5.11 | 4.27 | **4.48** |
|  | F12_p3 | |  |  | 0.834 | 0.916 | 0.923 | 1.07 | 1.11 | **0.971** |
| **x130151 strain** | |  | 3 successes  and 2 failures |  |  | | | | | |
|  | x130151**_** p1 | |  |  | 8.24 | 7.93 | 8.19 | 7.88 | 8.05 | **8.06** |
|  | x130151**_** p2 | |  |  | 3.67 | 4.02 | 3.94 | 3.76 | 3.81 | **3.84** |
| **x9 strain** | |  | 4 successes  and 1 failure |  |  | | | | | |
|  | x9_p1 | |  |  | 5.87 | 4.74 | 5.15 | 4.49 | 5.02 | **5.05** |
