# Supplementary material for: Characterization of Enterobacter cloacae complex clinical isolates: comparative genomics and the role of the efflux pump AcrAB-TolC over-expression and NDM-1 production
Source: Front Cell Infect Microbiol. 2025 Nov 7;15:1705370. doi: 10.3389/fcimb.2025.1705370 (PMC12635725; doi:10.3389/fcimb.2025.1705370)
Supplement: Supplementary Table 2 — Characterization of chromosomally encoded drug-resistance genes in strains F12, x9 and x230151. [file Table2.docx]

| **Table S2** Characterization of chromosomally encoded drug-resistance genes in strains F12, x9 and x230151 | | | | | | | | |
| --- | --- | --- | --- | --- | --- | --- | --- | --- |
| **Drug-resistance gene on chromosome**  (Sorted by gene location from smallest to largest) | | | **Model_**  **type** | **SNPs_** | **Other_**  **SNPs** | **Class of drug-resistance** | **Resistance mechanism** | **Antimicrobiol resistance**  **gene family** |
| **F12** | **x9** | **x230151** |  |  |  |  |  |  |
| *uhpT* | *uhpT* | *uhpT* | variant | E350Q | n/a | phosphonic acid antibiotic | antibiotic target alteration | antibiotic-resistant UhpT |
| *EF-Tu* | *EF-Tu* | *EF-Tu* | variant | R234F | n/a | elfamycin antibiotic | antibiotic target alteration | elfamycin resistant EF-Tu |
| *soxS* | *soxS* | *soxS* | Over  -expression | n/a | n/a | fluoroquinolone antibiotic; monobactam; carbapenem;  cephalosporin; glycylcycline; cephamycin; penam;  tetracycline antibiotic; rifamycin antibiotic; phenicol antibiotic; penem; disinfecting agents and antiseptics | antibiotic target alteration;  antibiotic efflux;  reduced permeability to antibiotic | ATP-binding cassette (ABC) antibiotic efflux pump;  major facilitator superfamily (MFS) antibiotic efflux pump; resistance-nodulation-cell division (RND) antibiotic efflux pump; General Bacterial Porin with reduced permeability to beta-lactams |
| *bla*_ACT-17_ | *bla*_ACT-17_ | *bla*_ACT-24_ | homolog | n/a | n/a | carbapenem; cephalosporin; cephamycin; penam | antibiotic inactivation | ACT beta-lactamase |
| *fosA2* | *fosA2* | *fosA2* | homolog | n/a | n/a | phosphonic acid antibiotic | antibiotic inactivation | fosfomycin thiol transferase |
| *leuO* | *leuO* | *leuO* | homolog | n/a | n/a | nucleoside antibiotic; disinfecting agents and antiseptics | antibiotic efflux | MFS antibiotic efflux pump |
| *PBP3* | *PBP3* | *PBP3* | variant | D350N, S357N | n/a | cephalosporin; cephamycin; penam | antibiotic target alteration | Penicillin-binding protein mutations conferring  resistance to beta-lactam antibiotics |
| *vanG* | *vanG* | *vanG* | homolog | n/a | n/a | glycopeptide antibiotic | antibiotic target alteration | glycopeptide resistance gene cluster; Van ligase |
| *acrB* | *acrB* | *acrB* | homolog | n/a | n/a | fluoroquinolone antibiotic; cephalosporin; glycylcycline;  penam; tetracycline antibiotic; rifamycin antibiotic;  phenicol antibiotic; disinfecting agents and antiseptics | antibiotic efflux | RND antibiotic efflux pump |
| *acrA* | *acrA* | *acrA* | homolog | n/a | n/a | fluoroquinolone antibiotic; cephalosporin; glycylcycline;  penam; tetracycline antibiotic; rifamycin antibiotic;  phenicol antibiotic; disinfecting agents and antiseptics | antibiotic efflux | RND antibiotic efflux pump |
| *ramA* | *ramA* | *ramA* | homolog | n/a | n/a | fluoroquinolone antibiotic; monobactam; carbapenem;  cephalosporin; glycylcycline; cephamycin; penam;  tetracycline antibiotic; rifamycin antibiotic; phenicol antibiotic; penem; disinfecting agents and antiseptics | antibiotic efflux;  reduced permeability  to antibiotic | RND antibiotic efflux pump |
| *mdfA* | *mdfA* | *mdfA* | homolog | n/a | n/a | tetracycline antibiotic; phenicol antibiotic;  disinfecting agents and antiseptics | antibiotic efflux | MFS antibiotic efflux pump |
| *msbA* | *msbA* | *msbA* | homolog | n/a | n/a | nitroimidazole antibiotic | antibiotic efflux | ABC antibiotic efflux pump |
| *kpnE* | *kpnE* | *kpnE* | homolog | n/a | n/a | acrolide antibiotic; aminoglycoside antibiotic; cephalosporin; tetracycline antibiotic; peptide antibiotic; rifamycin antibiotic; disinfecting agents and antiseptics | antibiotic efflux | small multidrug resistance (SMR) antibiotic efflux pump |
| *kpnF* | *kpnF* | *kpnF* | homolog | n/a | n/a | acrolide antibiotic; aminoglycoside antibiotic; cephalosporin; tetracycline antibiotic; peptide antibiotic; rifamycin antibiotic; disinfecting agents and antiseptics | antibiotic efflux | SMR antibiotic efflux pump |
| *marA* | *marA* | *marA* | homolog | n/a | n/a | fluoroquinolone antibiotic; monobactam; carbapenem;  cephalosporin; glycylcycline; cephamycin; penam;  tetracycline antibiotic; rifamycin antibiotic; phenicol antibiotic; penem; disinfecting agents and antiseptics | antibiotic efflux; reduced  permeability to antibiotic | RND antibiotic efflux pump; General Bacterial Porin  with reduced permeability to beta-lactams |
| *acrAB-tolC* | *acrAB-tolC* | *acrAB-tolC* | over-  expression | n/a | n/a | fluoroquinolone antibiotic; cephalosporin; glycylcycline;  penam; tetracycline antibiotic; rifamycin antibiotic; phenicol antibiotic; disinfecting agents and antiseptics | antibiotic target alteration;  antibiotic efflux | RND antibiotic efflux pump |
| *mdtB* | *mdtB* | *mdtB* | homolog | n/a | n/a | aminocoumarin antibiotic | antibiotic efflux | RND antibiotic efflux pump |
| *mdtC* | *mdtC* | *mdtC* | homolog | n/a | n/a | aminocoumarin antibiotic | antibiotic efflux | RND antibiotic efflux pump |
| *baeS* | *baeS* | *baeS* | over-  expression | n/a | n/a | aminoglycoside antibiotic; aminocoumarin antibiotic | antibiotic efflux | RND antibiotic efflux pump |
| *baeR* | *baeR* | *baeR* | over-  expression | n/a | n/a | aminoglycoside antibiotic; aminocoumarin antibiotic | antibiotic efflux | RND antibiotic efflux pump |
| *gyrA* | *-^#^* | *gyrA* | variant | S83F | S83F:2850, S83F:2403, S83F:2851 | fluoroquinolone antibiotic | antibiotic target alteration | fluoroquinolone resistant gyrA |
| *acrD* | *acrD* | *acrD* | homolog | n/a | n/a | aminoglycoside antibiotic | antibiotic efflux | RND antibiotic efflux pump |
| *-* | *-* | *oqxB* | homolog | n/a | n/a | fluoroquinolone antibiotic; glycylcycline; tetracycline antibiotic; diaminopyrimidine antibiotic; nitrofuran antibiotic | antibiotic efflux | RND antibiotic efflux pump |
| *adeF* | *adeF* | *-* | homolog | n/a | n/a | fluoroquinolone antibiotic; tetracycline antibiotic | antibiotic efflux | RND antibiotic efflux pump |
| *oqxA* | *oqxA* | *oqxA* | homolog | n/a | n/a | fluoroquinolone antibiotic; glycylcycline; tetracycline antibiotic; diaminopyrimidine antibiotic; nitrofuran antibiotic | antibiotic efflux | RND antibiotic efflux pump |
| *emrR* | *emrR* | *emrR* | homolog | n/a | n/a | fluoroquinolone antibiotic | antibiotic efflux | MFS antibiotic efflux pump |
| *emrB* | *emrB* | *emrB* | homolog | n/a | n/a | fluoroquinolone antibiotic | antibiotic efflux | MFS antibiotic efflux pump |
| *rsmA* | *rsmA* | *rsmA* | homolog | n/a | n/a | fluoroquinolone antibiotic; diaminopyrimidine antibiotic;  phenicol antibiotic | antibiotic efflux | RND antibiotic efflux pump |
| *parC* | *-* | *parC* | variant | S80I | n/a | fluoroquinolone antibiotic | antibiotic target alteration | fluoroquinolone resistant parC |
| *bacA* | *bacA* | *bacA* | homolog | n/a | n/a | peptide antibiotic | antibiotic target alteration | undecaprenyl pyrophosphate related proteins |
| *adeF* | *adeF* | *adeF* | homolog | n/a | n/a | fluoroquinolone antibiotic; tetracycline antibiotic | antibiotic efflux | RND antibiotic efflux pump |
| *EF-Tu* | *EF-Tu* | *EF-Tu* | variant | R234F | n/a | elfamycin antibiotic | antibiotic target alteration | elfamycin resistant EF-Tu |
| *crp* | *crp* | *crp* | homolog | n/a | n/a | macrolide antibiotic; fluoroquinolone antibiotic; penam | antibiotic efflux | RND antibiotic efflux pump |
| *ArnT* | *ArnT* | *-* | homolog | n/a | n/a | peptide antibiotic | antibiotic target alteration | pmr phosphoethanolamine transferase |
| *PmrF* | *PmrF* | *-* | homolog | n/a | n/a | peptide antibiotic | antibiotic target alteration | pmr phosphoethanolamine transferase |
| *^#^*-, no data | | | | | | | | |
