# Supplementary material for: Characterization of Enterobacter cloacae complex clinical isolates: comparative genomics and the role of the efflux pump AcrAB-TolC over-expression and NDM-1 production
Source: Front Cell Infect Microbiol. 2025 Nov 7;15:1705370. doi: 10.3389/fcimb.2025.1705370 (PMC12635725; doi:10.3389/fcimb.2025.1705370)
Supplement: Supplementary Table 3 — Primers for the acrAB-tolC gene in Enterobacter cloacae. [file Table3.docx]

| **Table S3** Primers for the *acrAB*-*tolC* gene in *Enterobacter cloacae* | | |
| --- | --- | --- |
| Primer | Sequence (5’-3’) | Product size (bp) |
| *acrA* | F: ACGTATTGGGCAATGACTGG | 191 |
|  | R: GGAGTCGCCGTCAATAGAAC |  |
| *acrB* | F: AAGAGCACGCATCACTACAC | 768 |
|  | R: CGCTTCGGACATCACGTAAA |  |
| *tolC* | F:CAGACGCTGATCCTCAATAC | 702 |
|  | R:TGCTGATGGAGGCGTTAATA |  |
