# Supplementary figures and images for: Characterization of Enterobacter cloacae complex clinical isolates: comparative genomics and the role of the efflux pump AcrAB-TolC over-expression and NDM-1 production
Source: Front Cell Infect Microbiol. 2025 Nov 7;15:1705370. doi: 10.3389/fcimb.2025.1705370 (PMC12635725; doi:10.3389/fcimb.2025.1705370)

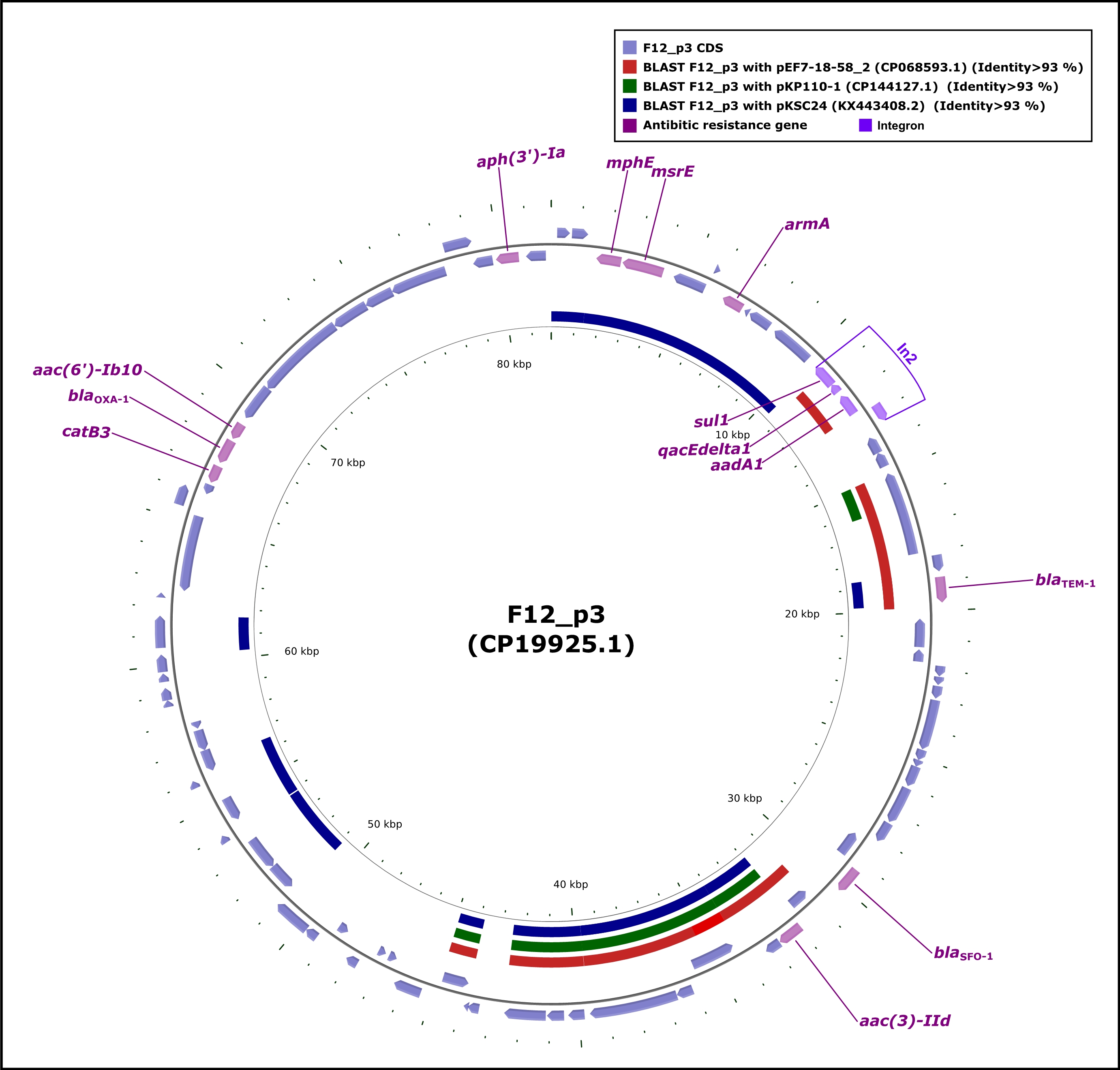

Supplement: Supplementary Figure 1 — Comparative plots of the complete F12_p3 plasmid against pEF7-18-58_2, pKP110-1, and pKSC24. The figure was produced with the R package genoPlotR v0.8.11, and illustrated manually using CGView v2.0.3. The locations of the antibiotic resistance genes were using Inkscape 0.48.1 (https://inkscape.org/en). High-identity alignment data for plasmid F12_p3 versus pEF7-18-58_2, pKP110-1, and pKSC24 were provided in Supplementary Materials (1-1) to (1-3). [file Image1.jpeg]

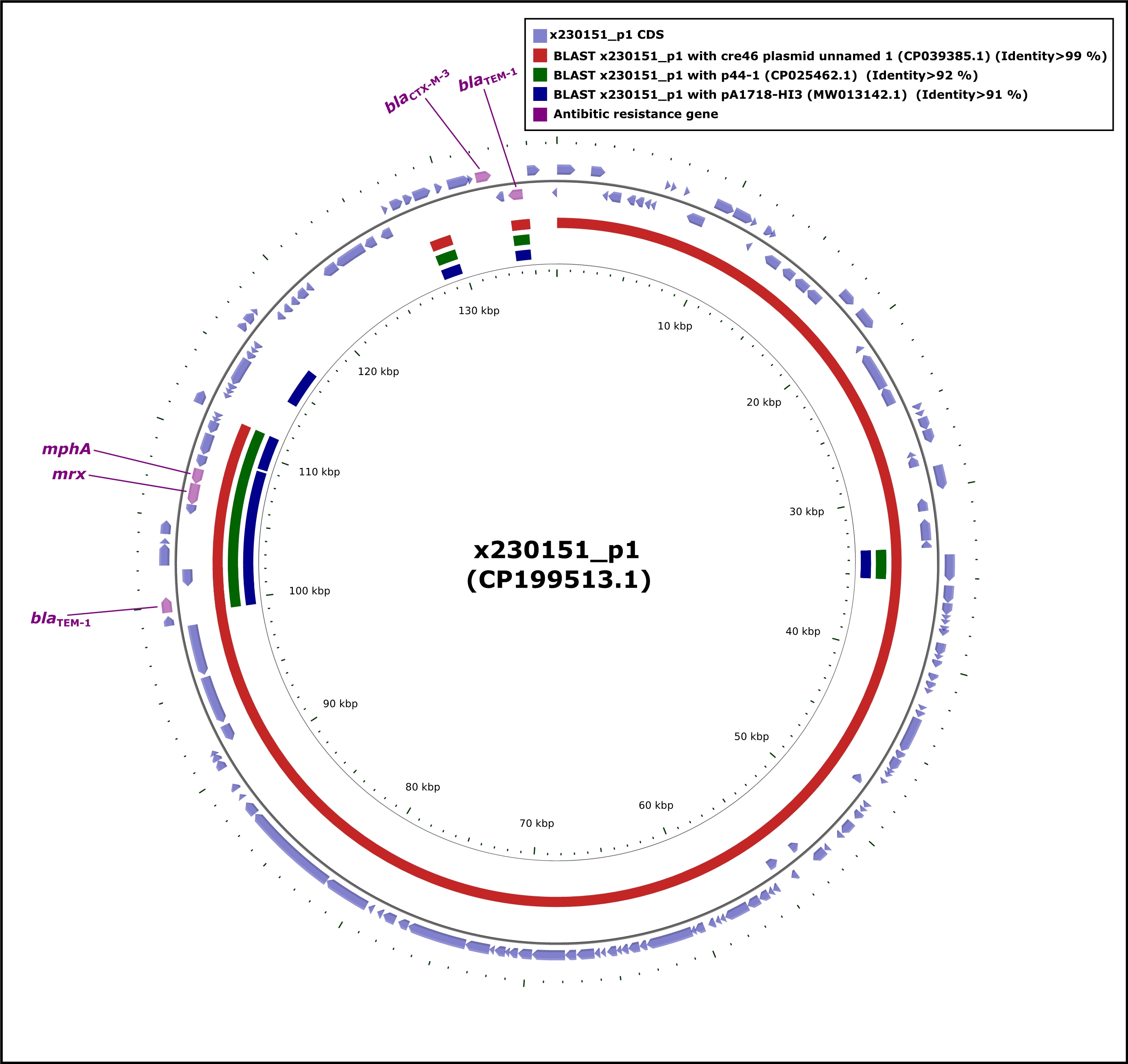

Supplement: Supplementary Figure 2 — Comparative diagram of the complete x230151_p1 plasmid against cre46 plasmid unnamed 1, p44-1, and pA1718-HI3. The figure was produced with the R package genoPlotR v0.8.11, and illustrated manually using CGView v2.0.3. The locations of the antibiotic resistance genes were using Inkscape 0.48.1 (https://inkscape.org/en). High-identity alignment data for plasmid x230151_p1 versus cre46 plasmid unnamed 1, p44-1, and pA1718-HI3 were provided in Supplementary Materials (1-4) to (1-6). [file Image2.jpeg]
