# Supplementary material for: Characterization of Enterobacter cloacae complex clinical isolates: comparative genomics and the role of the efflux pump AcrAB-TolC over-expression and NDM-1 production
Source: Front Cell Infect Microbiol. 2025 Nov 7;15:1705370. doi: 10.3389/fcimb.2025.1705370 (PMC12635725; doi:10.3389/fcimb.2025.1705370)
Supplement: Supplementary file 6 [file DataSheet1.zip › Supplementary Materials (1-3).docx]

| **Supplementary Material 1 - Pairwise high-identity alignment of plasmids x9_p1 and pCRE40_1 (CP074559.1)** | | | | | | | | |
| --- | --- | --- | --- | --- | --- | --- | --- | --- |
| **x9_p1**  **alignment (bp)** | **CP074559.1**  **alignment (bp)** | **Identity (%)** | **Coverage**  **(%)** | **Alignment**  **_length (bp)** | **Mismatches** | **Gap**  **_opens** | **E-value** | **Bit_score** |
| 1–12,204 | 166,060–178,263 | 100 | 100 | 12,204 | 0 | 0 | 0 | 22,537 |
| 13,521–14,954 | 178,256–179,689 | 100 | 100 | 1,434 | 0 | 0 | 0 | 2,649 |
| 14,955–18,267 | 138,027–134,715 | 100 | 100 | 3,313 | 0 | 0 | 0 | 6,119 |
| 21,125–22,181 | 4,885–5,941 | 95.36 | 100 | 1,057 | 49 | 0 | 0 | 1,681 |
| 27,307–30,258 | 90,868–93,819 | 99.97 | 100 | 2,952 | 1 | 0 | 0 | 5,446 |
| 28,460–30,237 | 126,247–124,470 | 90.38 | 100 | 1,778 | 171 | 0 | 0 | 2,337 |
| 32,251–44,858 | 90,880–78,274 | 99.96 | 100 | 12,608 | 4 | 1 | 0 | 23,254 |
| 39,800–40,805 | 132,875–131,870 | 100 | 100 | 1,006 | 0 | 0 | 0 | 1,858 |
| 58,421–60,622 | 178,870–181,070 | 98.14 | 100 | 2,203 | 38 | 3 | 0 | 3,838 |
| 65,372–69,466 | 188,025–192,119 | 94.99 | 100 | 4,095 | 205 | 0 | 0 | 6,427 |
| 69,455–70,487 | 300,799–299,766 | 99.42 | 100 | 1,034 | 5 | 1 | 0 | 1,875 |
| 79,135–80,334 | 66,858–65,658 | 91.86 | 100 | 1,204 | 91 | 7 | 0 | 1,674 |
| 80,332–88,086 | 139,553–147,307 | 99.87 | 100 | 7,755 | 10 | 0 | 0 | 14,266 |
| 88,079–106,063 | 148,076–166,059 | 99.99 | 100 | 17,985 | 1 | 1 | 0 | 33,200 |

| **Supplementary Material 2 - Pairwise high-identity alignment of plasmids x9_p1 and pSL131_IncA/C-IncX3 (MH105050.1)** | | | | | | | | |
| --- | --- | --- | --- | --- | --- | --- | --- | --- |
| **x9_p1**  **alignment (bp)** | **MH105050.1**  **alignment (bp)** | **Identity (%)** | **Coverage**  **(%)** | **Alignment**  **_length (bp)** | **Mismatches** | **Gap**  **_opens** | **E-value** | **Bit_score** |
| 1–14,958 | 181,383–166,425 | 99.99 | 100 | 14,959 | 0 | 1 | 0 | 27,617 |
| 33,352–38,078 | 96,414–101,140 | 99.96 | 100 | 4,727 | 2 | 0 | 0 | 8,719 |
| 38,078–39,807 | 102,467–104,195 | 99.83 | 100 | 1,730 | 2 | 1 | 0 | 3,177 |
| 39,774–44,860 | 112,647–107,561 | 99.9 | 100 | 5,087 | 5 | 0 | 0 | 9,367 |
| 44,038–46,250 | 106,544–104,332 | 100 | 100 | 2,213 | 0 | 0 | 0 | 4,087 |
| 71,290–80,334 | 216,895–207,851 | 100 | 100 | 9,045 | 0 | 0 | 0 | 16,704 |
| 80,332–106,063 | 207,115–181,384 | 100 | 100 | 25,732 | 0 | 0 | 0 | 47,519 |

| **Supplementary Material 3 - Pairwise high-identity alignment of plasmid x9_p1 and** **strain 65 chromosome (CP128442.1)** | | | | | | | | |
| --- | --- | --- | --- | --- | --- | --- | --- | --- |
| **x9_p1**  **alignment (bp)** | **CP128442.1**  **alignment (bp)** | **Identity (%)** | **Coverage**  **(%)** | **Alignment**  **_length (bp)** | **Mismatches** | **Gap**  **_opens** | **E-value** | **Bit_score** |
| 1–14,134 | 3,183,347–3,169,213 | 99.99 | 100 | 14,135 | 0 | 1 | 0 | 26,096 |
| 14,955–17,447 | 2,118,799–2,121,290 | 99.32 | 100 | 2,493 | 16 | 1 | 0 | 4,508 |
| 33,352–34,706 | 5,667,806–5,666,452 | 99.93 | 100 | 1,355 | 1 | 0 | 0 | 2,497 |
| 34,707–36,415 | 5,658,559–5,656,851 | 100 | 100 | 1,709 | 0 | 0 | 0 | 3,157 |
| 38,058–39,802 | 5,655,096–5,656,840 | 99.94 | 100 | 1,745 | 1 | 0 | 0 | 3,217 |
| 41,060–44,037 | 5,586,610–5,583,633 | 100 | 100 | 2,978 | 0 | 0 | 0 | 5,500 |
| 67,744–69,462 | 3,633,321–3,631,603 | 94.24 | 100 | 1,719 | 99 | 0 | 0 | 2,627 |
| 71,756–73,051 | 5,615,731–5,617,026 | 94.45 | 100 | 1,298 | 68 | 4 | 0 | 1,995 |
| 75,869–79,035 | 5,276,341–5,279,507 | 99.91 | 100 | 3,167 | 3 | 0 | 0 | 5,832 |
| 79,133–80,338 | 5,280,704–5,279,501 | 99.58 | 100 | 1,206 | 3 | 1 | 0 | 2,198 |
| 80,332–81,657 | 5,281,814–5,283,139 | 100 | 100 | 1,326 | 0 | 0 | 0 | 2,449 |
| 81877–83562 | 5,661,971–5,660,286 | 99.88 | 100 | 1,686 | 2 | 0 | 0 | 3,103 |
| 83,563–106,063 | 3,205,848–3,183,348 | 100 | 100 | 22,501 | 1 | 0 | 0 | 41,547 |
