# Supplementary material for: Characterization of Enterobacter cloacae complex clinical isolates: comparative genomics and the role of the efflux pump AcrAB-TolC over-expression and NDM-1 production
Source: Front Cell Infect Microbiol. 2025 Nov 7;15:1705370. doi: 10.3389/fcimb.2025.1705370 (PMC12635725; doi:10.3389/fcimb.2025.1705370)
Supplement: Supplementary file 6 [file DataSheet1.zip › Supplementary Materials (4-6).docx]

| **Supplementary Material 4 - Pairwise high-identity alignment of plasmids F12_p2 and F5111 (KU987453.1)** | | | | | | | | |
| --- | --- | --- | --- | --- | --- | --- | --- | --- |
| **F12_p2**  **alignment (bp)** | **KU987453.1**  **alignment (bp)** | **Identity (%)** | **Coverage**  **(%)** | **Alignment**  **_length (bp)** | **Mismatches** | **Gap**  **_opens** | **E-value** | **Bit_score** |
| 1–9,777 | 46,216–55,992 | 100 | 100 | 9,777 | 0 | 0 | 0 | 18,055 |
| 9,774–16,287 | 57,214–63,726 | 99.92 | 100 | 6,514 | 4 | 1 | 0 | 12,000 |
| 16,245–17,656 | 75,667–77,078 | 100 | 100 | 1,412 | 0 | 0 | 0 | 2,608 |
| 17,639–22,607 | 63,716–68,684 | 99.92 | 100 | 4,969 | 4 | 0 | 0 | 9,154 |
| 26,704–29,301 | 75,673–73,075 | 99.96 | 100 | 2,599 | 0 | 1 | 0 | 4,793 |
| 34,407–41,397 | 75,673–68,682 | 99.99 | 100 | 6,992 | 0 | 1 | 0 | 12,905 |
| 46,201–49,264 | 82,799–85,862 | 100 | 100 | 3,064 | 0 | 0 | 0 | 5,659 |
| 493,26–50,769 | 77,110–75,667 | 99.72 | 100 | 1,444 | 4 | 0 | 0 | 2,645 |
| 50,727–96,842 | 101–46,215 | 99.99 | 100 | 46,116 | 5 | 1 | 0 | 85,126 |

| **Supplementary Material 5 - Pairwise high-identity alignment of plasmids F12_p2 and pEA49-KPC (KU318419.1)** | | | | | | | | |
| --- | --- | --- | --- | --- | --- | --- | --- | --- |
| **F12_p2**  **Alignment (bp)** | **KU318419.1**  **alignment (bp)** | **Identity (%)** | **Coverage**  **(%)** | **Alignment**  **_length (bp)** | **Mismatches** | **Gap**  **_opens** | **E-value** | **Bit_score** |
| 1–13,424 | 41,371–27,943 | 99.95 | 100 | 13,429 | 2 | 1 | 0 | 24,755 |
| 18,828–29,301 | 19,895–9,423 | 99.49 | 100 | 10,475 | 50 | 3 | 0 | 19,047 |
| 33,717–46,200 | 12,710–227 | 100 | 100 | 12,484 | 0 | 0 | 0 | 23,054 |
| 46,476–49,375 | 90,351–87,452 | 100 | 100 | 2,900 | 0 | 0 | 0 | 5,356 |
| 50,727–56,319 | 87,462–81,865 | 99.77 | 100 | 5,601 | 2 | 6 | 0 | 10,261 |
| 56,346–96,842 | 81,867–41,372 | 99.95 | 100 | 40,505 | 3 | 17 | 0 | 74,672 |

| **Supplementary Material 6 - Pairwise high-identity alignment of plasmids** **F12_p2 and p72_4 (CP101558.1)** | | | | | | | | |
| --- | --- | --- | --- | --- | --- | --- | --- | --- |
| **F12_p2 alignment (bp)** | **CP101558.1**  **alignment (bp)** | **Identity (%)** | **Coverage**  **(%)** | **Alignment**  **_length (bp)** | **Mismatches** | **Gap**  **_opens** | **E-value** | **Bit_score** |
| 1–16,287 | 1–16,286 | 99.97 | 100 | 16,287 | 4 | 1 | 0 | 30,047 |
| 17,639–29,301 | 16,276–27,938 | 99.98 | 100 | 11,663 | 2 | 0 | 0 | 21,527 |
| 17,655–18,856 | 59,594–58,393 | 100 | 100 | 1,202 | 0 | 0 | 0 | 2,220 |
| 20,784–29,301 | 34,876–43,393 | 99.99 | 100 | 8,518 | 1 | 0 | 0 | 15,725 |
| 25,901–27,147 | 45,458–46,705 | 99.92 | 100 | 1,248 | 0 | 1 | 0 | 2,298 |
| 29,302–33,717 | 28,934–33,348 | 99.98 | 100 | 4,416 | 0 | 1 | 0 | 8,148 |
| 33,717–34,850 | 45,572–46,705 | 100 | 100 | 1,134 | 0 | 0 | 0 | 2,095 |
| 33,717–37,004 | 24,651–27,938 | 100 | 100 | 3,288 | 0 | 0 | 0 | 6,072 |
| 33,717–37,005 | 40,106–43,394 | 100 | 100 | 3,289 | 0 | 0 | 0 | 6,074 |
| 46,201–49,375 | 64,201–67,375 | 100 | 100 | 3,175 | 0 | 0 | 0 | 5,864 |
| 50,727–96,842 | 67,365–113,479 | 99.99 | 100 | 46,116 | 5 | 1 | 0 | 85,126 |
