# Supplementary material for: Characterization of Enterobacter cloacae complex clinical isolates: comparative genomics and the role of the efflux pump AcrAB-TolC over-expression and NDM-1 production
Source: Front Cell Infect Microbiol. 2025 Nov 7;15:1705370. doi: 10.3389/fcimb.2025.1705370 (PMC12635725; doi:10.3389/fcimb.2025.1705370)
Supplement: Supplementary file 6 [file DataSheet1.zip › Supplementary Materials (7-9).docx]

| **Supplementary Material 7 - Pairwise high-identity alignment of plasmids x230151_p2 and CRECL11 unnamed1** **(CP166889.1)** | | | | | | | | |
| --- | --- | --- | --- | --- | --- | --- | --- | --- |
| **x230151_p2 alignment (bp)** | **CP166889.1**  **alignment (bp)** | **Identity (%)** | **Coverage**  **(%)** | **Alignment**  **_length (bp)** | **Mismatches** | **Gap**  **_opens** | **E-value** | **Bit_score** |
| 1–10,399 | 1–10,399 | 100 | 100 | 10,399 | 0 | 0 | 0 | 19,204 |
| 10,391–23,831 | 14,443–27,883 | 100 | 100 | 13,441 | 0 | 0 | 0 | 24,821 |
| 28,297–33,982 | 37,673–31,988 | 100 | 100 | 5,686 | 0 | 0 | 0 | 10,501 |
| 32,243–33,376 | 39,084–37,951 | 100 | 100 | 1,134 | 0 | 0 | 0 | 2,095 |
| 45,027–48,547 | 35,796–39,316 | 99.66 | 100 | 3,523 | 8 | 4 | 0 | 6,436 |
| 47,182–48,315 | 32,594–33,727 | 100 | 100 | 1,134 | 0 | 0 | 0 | 2,095 |
| 49,395–53,368 | 40,707–44,680 | 99.92 | 100 | 3,974 | 3 | 0 | 0 | 7,323 |
| 53,351–54,721 | 29,645–28,275 | 93.29 | 100 | 1,371 | 92 | 0 | 0 | 2,023 |
| 54,695–134,108 | 44,680–124,093 | 100 | 100 | 79,414 | 3 | 0 | 0 | 146,600 |

| **Supplementary Material 8 - Pairwise high-identity alignment of plasmids x230151_p2 and pECC-143-1 (CP143709.1)** | | | | | | | | |
| --- | --- | --- | --- | --- | --- | --- | --- | --- |
| **x230151_p2**  **alignment (bp)** | **CP143709.1**  **alignment (bp)** | **Identity (%)** | **Coverage**  **(%)** | **Alignment**  **_length (bp)** | **Mismatches** | **Gap**  **_opens** | **E-value** | **Bit_score** |
| 1–23,831 | 1–23,831 | 100 | 100 | 23,831 | 1 | 0 | 0 | 44,003 |
| 28,297–33,982 | 33,621–27,936 | 100 | 100 | 5,686 | 0 | 0 | 0 | 10,501 |
| 32,243–33,376 | 35,032–33,899 | 100 | 100 | 1,134 | 0 | 0 | 0 | 2,095 |
| 45,027–48,547 | 31,744–35,264 | 99.66 | 100 | 3,523 | 8 | 4 | 0 | 6,436 |
| 47,182–48,315 | 28,542–29,675 | 100 | 100 | 1,134 | 0 | 0 | 0 | 2,095 |
| 49,395–134,108 | 36,655–121,368 | 99.89 | 100 | 84,714 | 97 | 0 | 0 | 155,900 |
| 53,351–54,721 | 25,593–24,223 | 93.29 | 100 | 1,371 | 92 | 0 | 0 | 2,023 |

| **Supplementary Material 9 - Pairwise high-identity alignment of plasmids x230151_p2 and pECC-102-1 (CP143730.1)** | | | | | | | | |
| --- | --- | --- | --- | --- | --- | --- | --- | --- |
| **x230151_p2**  **alignment (bp)** | **CP143730.1**  **alignment (bp)** | **Identity (%)** | **Coverage**  **(%)** | **Alignment**  **_length (bp)** | **Mismatches** | **Gap**  **_opens** | **E-value** | **Bit_score** |
| 1–23,831 | 1–23,831 | 100 | 100 | 23,831 | 1 | 0 | 0 | 44,003 |
| 28,297–33,982 | 33,621–27,936 | 100 | 100 | 5,686 | 0 | 0 | 0 | 10,501 |
| 32,243–33,376 | 35,032–33,899 | 100 | 100 | 1,134 | 0 | 0 | 0 | 2,095 |
| 45,027–48,547 | 31,744–35,264 | 99.66 | 100 | 3,523 | 8 | 4 | 0 | 6,436 |
| 47,182–48,315 | 28,542–29,675 | 100 | 100 | 1,134 | 0 | 0 | 0 | 2,095 |
| 49,395–53,368 | 36,655–40,628 | 99.92 | 100 | 3,974 | 3 | 0 | 0 | 7,323 |
| 53,351–54,721 | 25,593–24,223 | 93.29 | 100 | 1,371 | 92 | 0 | 0 | 2,023 |
| 54,695–134,108 | 40,628–120,041 | 100 | 100 | 79,414 | 3 | 0 | 0 | 146,600 |
